# Unravelling the Atomic Structure of a Metal‐Covalent Organic Framework Assembled from Ruthenium Metalloligands

**DOI:** 10.1002/adma.202502155

**Published:** 2025-02-19

**Authors:** Seán Hennessey, Roberto González‐Gómez, Nicolás Arisnabarreta, Anna Ciotti, Jing Hou, Nadezda V. Tarakina, Andrey Bezrukov, Kunal S. Mali, Michael Zaworotko, Steven De Feyter, Max García‐Melchor, Pau Farràs

**Affiliations:** ^1^ School of Biological and Chemical Sciences Energy Research Centre Ryan Institute University of Galway University Road Galway H91 TK33 Ireland; ^2^ Division of Molecular Imaging Photonics Department of Chemistry KU Leuven Celestijnenlaan 200F Leuven 3001 Belgium; ^3^ School of Chemistry CRANN and AMBER Research Centres Trinity College Dublin College Green Dublin D02 PN40 Ireland; ^4^ Department of Colloid Chemistry Max Planck Institute of Colloids and Interfaces Research Campus Golm Am Mühlenberg 1 14476 Potsdam Germany; ^5^ Bernal Institute University of Limerick Limerick V94 T9PX Ireland; ^6^ Center for Cooperative Research on Alternative Energy (CIC EnergiGUNE) Basque Research and Technology Alliance (BRTA) Alava Technology Park, Albert Einstein 48 Vitoria‐ Gasteiz 01510 Spain; ^7^ IKERBASQUE Basque Foundation for Science Plaza de Euskadi 5 Bilbao 48009 Spain

**Keywords:** 2D frameworks, DFT calculations, metal‐covalent organic framework, metalloligands, photoactive materials

## Abstract

Covalent and metal‐organic frameworks (COFs and MOFs) have shown great promise in light‐driven processes mainly due to their ligand‐to‐metal charge‐separation properties, as well as having access to a diverse range of photoactive metalloligands and organic linkers. However, both frameworks present individual drawbacks that can potentially be avoided by combining both systems (metal and covalent) to produce metal‐covalent organic frameworks (MCOFs), exhibiting the advantages of both material types. Yet, due to their poor crystallinity, the understanding of the structure‐properties relation of MCOFs remains unclear. Herein, we report photoactive linkers in the form of a [Ru(tpy)_2_]^2+^ (tpy: 2,2′,6,2″‐terpyridine) complex which covalently binds to a luminescent pyrene core to yield a new, photoactive Schiff‐base MCOF. The structure, thermal, electronic, and optical properties of this novel material have been exhaustively characterized by a wide range of microscopy, spectroscopic, and computational methods. This combined experimental and computational work represents a significant step toward the fundamental understanding of the photoactive units within the framework, their hierarchical arrangement and interactions with substrates, which is essential for the future design of efficient photocatalytic materials.

## Graphical abstract

Metal‐covalent organic frameworks (MCOFs) are novel porous materials that exhibit the advantages of covalent and metal‐organic frameworks. However, it is extremely difficult to resolve their atomic structure and better understand their structure‐properties relation. This work utilizes a synergistic combination of advanced microscopy, spectroscopic, and computational methods to unravel the atomic mapping of a photoactive ruthenium‐MCOF.

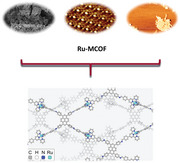

## 1 Introduction

Inspired by natural light‐harvesting systems, a range of artificial photoactive constituents have been assembled to mimic the complex antenna protein units present within the photosynthetic process and reduce the instabilities of molecular approaches.^[^ ^1^ ^]^ One explored domain of such light‐harvesting systems is that of porous materials, most notably metal‐organic frameworks (MOFs) and covalent organic frameworks (COFs). Their thermal and chemical stability combined with their porous nature have led to rapid growth in different applications ranging from gas separation and storage to heterogeneous catalysis.^[^ ^2^, ^3^, ^4^, ^5^, ^6^ ^]^ In the case of MOFs, the homogeneous distribution of metals in the frameworks has seen implementation in various forms of catalysis,^[^ ^7^, ^8^, ^9^ ^]^ although, they have not been fully exploited as photocatalysts or light‐harvesting materials, primarily due to their relatively fast electron‐hole recombination process and fairly poor long‐term stability.^[^ ^10^ ^]^ On the other hand, COFs exhibit excellent electron transport due to their conjugation and 2D stacked arrangement. As a result, these materials have demonstrated significant photophysical and luminescent properties, being applied in chemical sensing and photocatalysis.^[^ ^11^, ^12^ ^]^ However, the number of building blocks available for the assembly of photoactive COFs is currently limited, and when irradiated, the competing non‐radiative pathways that lead to quenching are abundant.^[^ ^13^ ^]^

Aimed at overcoming the individual limitations of MOFs and COFs, an understudied area of research in this field is the combination of such systems to develop 2D MCOFs.^[^ ^14^ ^]^ In particular, the poor long‐term stability of MOFs can be improved by the introduction of strong covalent linkages in the metal‐covalent organic framework (MCOF) material, while the absence of metal sites in COFs, which limits their applicability, can be addressed by installing metal ions into the covalently linked structure. In a variety of examples, this mixed architectural approach has been shown to produce materials that retain porosity, improve stability, and maintain some degree of crystallinity while preserving their tunability.^[^ ^15^ ^]^ Furthermore, the electronic communication between the metal centers, as a result of their covalent interactions, gives promise for these materials to be utilized in many practical applications, including sensing, catalysis, and light harvesting.^[^ ^16^, ^17^, ^18^, ^19^ ^]^

Despite the great potential of MCOFs materials, there is a notable gap in the fundamental understanding of their structural properties and formation, which hampers their widespread applicability. Recently, great strides have been made in the areas of crystallography and microscopy to probe the real‐time dynamics and structural determination of low crystalline frameworks.^[^ ^20^, ^21^, ^22^, ^23^ ^]^ The crystallinity of MCOFs is, in general, significantly reduced compared to other ordered porous materials, which makes it very challenging to obtain reliable information about their nanostructure. It is envisaged that a well‐mapped atomic structure of COF‐like materials will provide paramount information on its interactions with target substrates toward promoting selective catalysis, however, resolving their structure is a demanding task.^[^ ^24^ ^]^ Nevertheless, without experimental evidence to support their atomic arrangement, key parameters such as the interaction between different photoactive components within the framework are poorly understood, which ultimately have a direct impact on their potential applications.

Numerous reported light‐harvesting MOFs and COFs incorporate luminescent building blocks such as pyrene and triphenylbenzene derivatives due to their planar shape, facile functionalization, well‐understood emission properties, and long‐lived excited states.^[^ ^13^, ^25^, ^26^, ^27^, ^28^ ^]^ Ru‐polypyridine complexes, acting as metalloligands, have been widely incorporated in photoactive coordination polymers and frameworks because of their excellent light‐harvesting and photoredox properties.^[^ ^29^ ^]^ However, the commonly used [Ru(bpy)_3_]^2+^ (bpy: 2,2′‐bipyridine) moiety, while effective, can lead to mixtures of enantiomers and diastereomers that may promote undesirable back electron transfer reactions.^[^ ^30^ ^]^

An alternative Ru‐polypyridine light‐harvesting species are the [Ru(tpy)_2_]^2+^ complexes, which adopt a linear geometry upon functionalization at the *para*‐position of the central pyridine ring, making them very attractive as metalloligands in coordination polymers as they resemble the typical organic linkers used in MOFs and COFs. Although relatively few examples exist, when incorporated into photoactive frameworks, [Ru(tpy)_2_]^2+^ complexes exhibit exceptional potential for light‐harvesting, photovoltaics, and photocatalysis.^[^ ^31^, ^32^, ^33^, ^34^, ^35^, ^36^ ^]^

**Figure 1:**
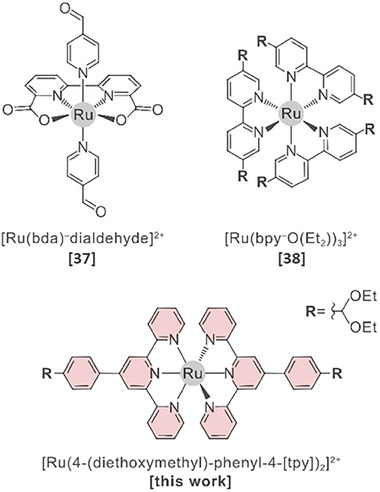
Examples of Ru‐based metalloligands reported in the literature used for the synthesis of MCOF systems, with references given in brackets.

More recently, [Ru(bpy)_3_]^2+^ derivatives have been introduced into MCOF systems (**Figure** **1**) and tested for photochemical water oxidation. Würthner et al. successfully incorporated a [Ru(bda)]‐based (bda: 2,2′‐bipyridine‐6,6′‐dicarboxylate) dialdehyde into a covalently linked interpenetrated MCOF.^[^ ^37^ ^]^ The degree of crystallinity resulted in a great enhancement in the catalytic activity, outperforming its amorphous polymer counterpart, both in terms of stability and activity. This was achieved by using a flexible tetra‐(4‐anilyl)methane linker that provided enough room to obtain a crystalline interpenetrated framework. In a similar vein, Zhou et al. have recently incorporated a [Ru(bpy)_3_]^2+^‐based moiety into a 3D MCOF containing luminophore (4,4′,4′′,4′′′‐(ethene‐1,1,2,2‐tetrayl)tetraaniline), which displayed a well‐defined (100) peak at 5° but the other peaks had significantly lower intensity.^[^ ^38^ ^]^ This framework displayed excellent performance for visible‐light‐driven hydrogen evolution and photoredox organic transformations, highlighting the enormous potential of these porous systems in photocatalytic and light‐harvesting applications.

In other examples of Ru‐based MCOFs, the elucidation of the material arrangement has not been achieved through crystal structure determination, which is notoriously difficult in the case of COFs.^[^ ^39^, ^40^ ^]^ While structural insights can be obtained through a combination of a variety of techniques, including powder X‐ray diffraction (PXRD), solid‐state nuclear magnetic resonance (ssNMR), and infrared spectroscopy (IR), corroboration of the atomic arrangement within the structure often requires modeling studies due to the complexity of the reticular architectures.

Planar 2D COF systems have been extensively studied using advanced microscopy techniques to great effect.^[^ ^41^, ^42^, ^43^, ^44^ ^]^ However, to the best of our knowledge, structural characterization of MCOF systems at the atomic level is lacking, in great part due to the structural damage undergone with high‐intensity beams, as with high‐resolution transmission electron microscopy (HR‐TEM). In addition, the octahedral geometry of species like [Ru(tpy)_2_]^2+^ linkers results in an out‐of‐plane topology on top of the complex stacking effects inherent to the material. These quasi‐2D materials are, therefore, challenging to fully characterize through scanning probe microscopy techniques such as scanning tunneling microscopy (STM) and atomic force microscopy (AFM). Accordingly, a complete understanding of the structural behavior, i.e., crystallinity, stacking effects, and morphology at the atomic scale, would be of great significance to the field, guiding both the structural design and evaluation of the photophysical properties of future 2D porous materials. In the case of COFs, the use of STM alongside computational modeling has shown the complexity involved in elucidating the structure of planar systems,^[^ ^45^ ^]^ while this combination of techniques has also been used to deduce lattice structures in other complex 2D materials.^[^ ^46^ ^]^

Herein, we report a [Ru(tpy)_2_]^2+^ derivative metalloligand decorated with terminal acetal groups, which has been covalently linked to an amine‐functionalized pyrene core, 4,4′,4′′,4′′′‐(pyrene‐1,3,6,8‐tetrayl)tetraaniline (PyTTA) to produce a novel photoactive Schiff‐base MCOF. Through the combination of advanced microscopy techniques and computational methodologies, we have elucidated the atomic structure of this novel MCOF, providing evidence of the formation of a well‐ordered semi‐planar 2D framework, arranged at the nanoscale as a bilayer structure with shifted AB‐stacking interactions. This work lays the foundation for deeper understanding of the structure‐property relationships in materials, providing crucial insights for the future development of more efficient photoactive materials.

## 2 MCOF Design and Characterization

The chelating ligand 4‐(diethoxymethyl)‐phenyl‐4‐[2,2′;6′,2′’‐terpyridine] (1) was synthesized by following a procedure for the synthesis of phenyl‐substituted terpyridines reported elsewhere,^[^ ^47^ ^]^ and characterized by ^1^H‐NMR, ^13^C‐NMR, and mass spectrometry (MS) (Figures S1–S3, Supporting Information). The acetal‐functionalized terpyridine could be readily converted into the free aldehyde, 4‐[2,2′;6′,2′']‐terpyridin‐4‐benzaldehyde (2), via acid reflux (Figures S4–S6, Supporting Information); however, complexation of 2 with the ruthenium precursor, [Ru(DMSO)_4_Cl_2_], led to an inseparable mixture of ruthenium products.

**Scheme 1:**
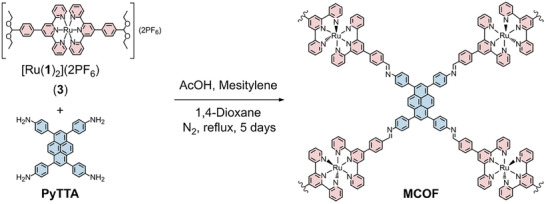
Synthesis of the Ru‐pyrene MCOF. Curly lines indicate the continuation of the ordered framework connecting to other pyrene units.

Taking inspiration from Wang et al.,^[^ ^48^ ^]^ who showcased the one‐pot direct condensation of acetal‐adorned ligands into imine‐linked COFs, the acetal‐decorated ruthenium complex [Ru(1)_2_](2PF_6_) (3), shown in Scheme 1, was synthesized directly from ligand 1. Complex 3 was then isolated by successive recrystallizations of acetone:diethyl ether mixture, and fully characterized by ^1^H‐NMR, ^13^C‐NMR, MS, ultraviolet‐visible spectroscopy (UV–vis), and IR (Figures S7–S11, Supporting Information). By using acidic media to produce the aldehyde in situ, the metalloligand 3 and PyTTA were heated at reflux in 1,4‐dioxane under nitrogen atmosphere. As a result of the octahedral geometry of the [Ru(tpy)_2_]^2+^‐based metalloligand, which limits potential π–π stacking, as well as the substantial pore size of the material, developing single crystals was particularly challenging. This was evident with the appearance of a dark red powder within three hours, which was left further for five days to promote the growth of an ordered network, as shown in **Scheme** **1**. The precipitate was collected by filtration and washed extensively with tetrahydrofuran (THF), methanol (CH_3_OH), and acetonitrile (CH_3_CN) to ensure the complete removal of starting materials. The cleaning step was further facilitated by sonication of the material in CH_3_OH for three hours and additional filtration. After drying, IR analyses (Figures S11 and S12, Supporting Information) confirmed that the main vibrational bands of both 3 and PyTTA were present in the Ru‐pyrene MCOF, while the ν_N‐H_ bands at ca. 3340 and 3370 cm^−1^ of PyTTA were not detected, indicating no unreacted amine groups remained in the Ru‐pyrene MCOF.

The distinctive imine stretching vibration, ν_C═N_, typically present at ca. 1680 cm^−1^, could not be resolved due to the overlap with the terpyridine vibrational bands in 3, which cover the range between 1550−1700 cm^−1^, further proof is provided later through DFT. To circumnavigate this issue, solid‐state ^13^C‐NMR was performed on the Ru‐pyrene MCOF to confirm the presence of the imine carbon (Figure S13, Supporting Information). The recorded spectrum showed the presence of the imine signal C═N in the range 151–157 ppm, proving the bonding between the in situ synthesized aldehyde and the amine of the pyrene unit. An additional signal observed at ca. 194 ppm was attributed to residual terminal aldehyde groups at the edges of the framework. The relation of Ru‐terpyridine units to pyrene building blocks was further confirmed by inductively coupled plasma‐optical emission spectrophotometer (ICP‐OES) analysis, finding that the % wt_Ru_ content in the MCOF was 7.47% (Figure S14, Supporting Information). The obtained value is in good agreement with the predicted weight of ruthenium in the idealized 1:1 Ru‐pyrene framework (7.61%), corroborating the formation of the network and the ratio between the different building blocks.

**Figure 2:**
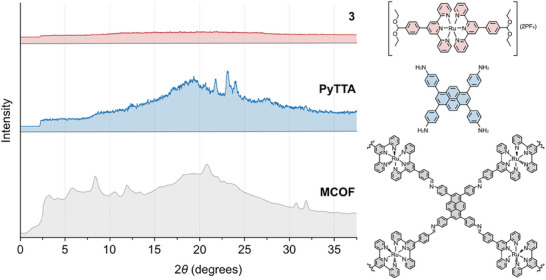
Recorded PXRD patterns of 3 (red), PyTTA (blue), and the Ru‐pyrene MCOF (grey).

The crystallinity of the Ru‐pyrene MCOF was investigated further by PXRD (**Figure** **2**; Figures S15 and S16, Supporting Information), as single crystals of the material could not be isolated. The diffraction pattern presented in Figure 2 shows a semi‐crystalline material with an array of new peaks appearing between 3 and 25 (2θ) degrees, in striking contrast to the poor crystallinity of PyTTA and the relative amorphous nature of 3. The peak broadening observed in the PXRD spectrum of the Ru‐pyrene MCOF was attributed to the small sizes of the crystallites (0.2–3.0 µm^2^), as microscopy measurements later corroborated. In addition, the poor resolution of the peaks between 15–25 (2θ) degrees in the MCOF sample is owed to the polyimide sample holder.^[^ ^49^ ^]^ Moreover, to stress the data at low‐angle X‐ray diffraction, small‐angle X‐ray scattering (SAXS) was performed, revealing the same diffraction pattern profile as PXRD, and non‐new relevant signals were observed (Figure S17, Supporting Information).

After confirming the formation and high‐level structure of the Ru‐pyrene MCOF, its thermal stability was investigated by thermal gravimetric analysis‐differential scanning calorimetry (TGA‐DSC) and compared against its molecular building blocks (Figure S18, Supporting Information). Robust thermal stability of the Ru‐pyrene MCOF was observed up to 380 °C, with the material preserving 90% of its initial weight. Weight loss below 100 °C was attributed to CH_3_OH absorbed in the material, indicating already a certain degree of porosity, as expected for the MCOF. Between 380–425 °C, a dramatic weight loss of 80% was measured; this significant drop within such a narrow temperature range is suggestive of the sudden decomposition of the linkages in the material, resulting in the collapse of the ordered framework. Further evidence of this is supported by the appearance of only one thermal transition throughout that temperature range, implying that the breakdown of the MCOF is due to the rapid degradation of the Ru metalloligand, while the pyrene unit, a more thermally stable moiety, initiates to decompose above 425 °C (Figure S19, Supporting Information).

Brunauer‐Emmett‐Teller (BET) analysis of the Ru‐pyrene MCOF provided a strong indication of porosity in the framework, with a calculated BET surface area from CO_2_ at 195 K measurement of 88.96 ± 3.49 m^2^ g^−1^ (Figure S20, Supporting Information). This feature is encouraging for further applications, such as catalysis, due to the possible incorporation of active molecules into the pores of the material, although, the porosity is relatively low compared to other Ru‐MCOF systems which have shown to be in the range between 120–235 m^2^ g^−1^.^[^ ^38^ ^]^

The Ru‐pyrene MCOF was next characterized electrochemically to confirm the presence of the metalloligand by comparing the Ru^III/II^ redox peaks with the free complex in solution, as well as to shed light on the framework's stability in harsh oxidative environments. Electrochemical measurements were carried out by drop casting the Ru‐pyrene MCOF with Nafion onto a glassy carbon electrode and collecting 100 cyclic voltammetry (CV) scans within a potential window from +0.50 to +1.60 V_SCE_ at a scan rate of 0.1 V s^−1^. The presence of the reversible Ru^III/II^ couple at 1.24 V_SCE_ was still evident after 100 scans (Figure S21, Supporting Information) with no sign of degradation, showcasing the redox stability of the material. The retention of redox activity is very promising for potential uses in photovoltaic and photocatalytic applications, as the redox couple is essential in these systems.

**Figure 3:**
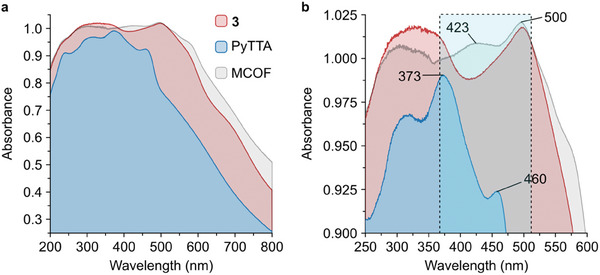
Optical characterization of the Ru‐pyrene MCOF. a) UV–Vis‐DRS of 3 (red), PyTTA (blue), and Ru‐pyrene MCOF (grey). b) Zoom‐in spectra with the main band shifts labeled.

To explore the potential of the Ru‐pyrene MCOF as a light‐harvesting material, we proceeded to characterise the photophysical properties of the system. Due to its insolubility in traditional organic solvents, solid‐state UV‐Vis diffuse reflectance spectroscopy (UV–Vvis‐DRS) measurements were carried out (**Figure** **3**).

The main band observed at ca. 500 nm for both the Ru‐metalloligand 3 (red profile) and Ru‐pyrene MCOF (grey profile) can be attributed to the metal‐ligand charge transfer within the Ru‐terpyridine unit and is indicative of the presence of unaltered [Ru(tpy)_2_]^2+^ moieties in the framework. Furthermore, the band appearing at ca. 423 nm evidenced the presence of the pyrene building block in the framework, as the isolated PyTTA ligand (blue profile) presents distinct peaks at ca. 373 and 460 nm, which typically shift when the ligand is coordinated due to the framework formation preventing the self‐aggregation of the ligand.^[^ ^50^ ^]^ Additionally, the fluorescence properties of the material were investigated by emission spectroscopy using poly(methyl methacrylate) (PMMA) films on quartz slides. At an excitation wavelength of 350 nm, the MCOF film (Figure S22, Supporting Information) displays a strong emission profile with a significant blue shift in the emission spectra (465→451 nm) in comparison with that of PyTTA, once again confirming the presence of pyrene in the material.

From the photophysical results, it can be demonstrated that by integrating these two photoactive components into a single framework, the window of their light‐harvesting features can be expanded while retaining their chemical and optical properties. The photophysical characteristics of the material, in combination with its thermal and chemical stability, highlight the excellent potential of the Ru‐pyrene MCOF in light‐harvesting applications.

## 3 Micro‐ to Atomic‐Scale Structural Characterization

The results described so far demonstrate that 3 and PyTTA were successfully integrated into the Ru‐pyrene MCOF structure, that the optical and electrochemical properties of the Ru complex 3 are preserved, and that the resulting material is semi‐crystalline. However, the morphology and structure at the micro and atomic scale, as well as the details of how the individual building blocks are connected and any stacking effects are lacking. To shed light on these matters at the micro‐scale, a range of advanced microscopy techniques were employed, as described below.

**Figure 4:**
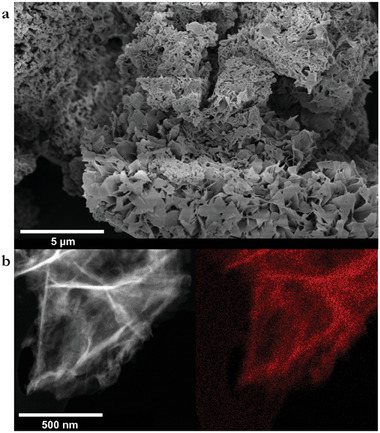
Scanning Electron Microscopy images of the MCOF. a) SEM micrograph of the Ru‐pyrene MCOF. b) EDX mapping of the Ru content (red) on the Ru‐pyrene MCOF.

Firstly, scanning electron microscopy (SEM) revealed the growth of a consistent flake‐like material (**Figure** **4**a; Figure S23, Supporting Information), ranging from 0.5 to 10.0 µm in length. The large size of these units compared to that of the material after sonication, shown by atomic force microscopy (AFM) and transmission electron microscopy (TEM) (Figures S26–S28, Supporting Information), indicated that the flake structures are formed through the aggregation of small units of the Ru‐pyrene MCOF. In addition, a small number of spherical particles of 0.5–0.8 µm in diameter were observed, suggesting partial growth of the network or evolution to a different morphological form out of the flakes. Energy dispersive X‐ray (EDX) and elemental mapping analyses on the Ru‐pyrene MCOF (Figure 4b; Figures S24 and S25, Supporting Information) showed a consistent peak corresponding to ruthenium in all measurements undertaken, as well as a homogeneous distribution of the metal throughout the framework.

Further TEM measurements performed on the bulk framework revealed clusters of several hundred nanometres in length, in line with AFM analysis (Figures S26 and S28a, Supporting Information). To determine the morphology of the Ru‐pyrene MCOF at a smaller scale, the framework was exfoliated by sonication in ethanol for six hours at 50 °C to break down the large aggregates into smaller units for further analysis. TEM and AFM images after exfoliation clearly indicated that the flake structures found in the bulk framework broke down into smaller units (Figures S27 and S28b, Supporting Information), reinforcing the hypothesis that the flakes are aggregates formed of well‐ordered nanoclusters of the Ru‐pyrene MCOF.

**Figure 5:**
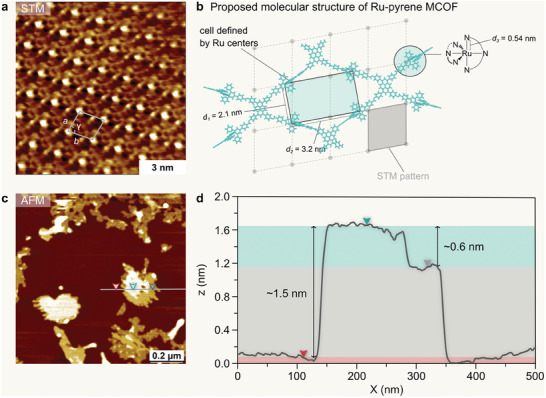
Scanning probe microscopies characterization of the Ru‐pyrene MCOF. a) High‐resolution STM image showing the Ru‐pyrene MCOF imaged at the heptanoic acid/HOPG interface. The measured distances and angle between repeating units obtained from calibrated STM images are: *a* = 1.7 ± 0.1 nm; *b* = 1.8 ± 0.1 nm; γ = 80 ± 3°. Imaging parameters: *V*_bias_ = –0.4 V, *I*_set_ = 150 pA. b) Molecular models of the Ru‐pyrene MCOF and Ru‐metalloligand 3 (inset). Relevant intramolecular and intralayer distances between Ru atoms are given in nm. The STM pattern is overlaid in grey. c) AFM image showing the layered morphology of the Ru‐pyrene MCOF. d) Line profile across one of the islands in (c) showing the step height of ca. 0.6 nm.

The nanoscale structure of the Ru‐pyrene MCOF was next investigated via STM. Deposition of the ruthenium complex 3 and the PyTTA monomers at the heptanoic acid/ highly oriented pyrolytic graphite (HOPG) interface in the presence of solid particles of 3 (see Supporting Information for details) led to the formation of isolated 2D crystallites of the Ru‐pyrene MCOF ranging in size from 10^2^ to 10^3^ nm^2^ (Figure S29, Supporting Information). It is worth noting that no molecular adlayers were observed after individual deposition of single monomers, indicating that neither of the building blocks was able to self‐assemble at the solid/liquid interface under the investigated conditions. Conversely, the appearance of molecular adlayers was confirmed by high‐resolution STM in the presence of both monomers, as well as solid particles of the MCOFs. The incorporation of solid crystallites of 3 on the HOPG surface, previously covered with a pre‐mixture of monomers, acts as a seed for the growth and successful molecular‐scale STM imaging of the MCOF recently reported for other imine‐based 2D‐COF systems.^[^ ^51^ ^]^ **Figure** **5**a shows a high‐resolution STM image revealing an oblique crystalline lattice. Surprisingly, the measured distances between bright spots (*a* = 1.7 ± 0.1 nm; *b* = 1.8 ± 0.1 nm) are significantly smaller than the expected intralayer Ru–Ru distances in the Ru‐pyrene MCOF structure (i.e.*, d_1_* = 2.1 nm; *d_2_* = 3.2 nm). This discrepancy can be understood by considering the formation of a bilayer system of the Ru‐pyrene MCOF at the solution‐solid interface. The boundary of the domain of the Ru‐pyrene MCOF, as well as the topology of the framework at a larger scale, can also be visualized at the heptanoic acid/HOPG interface using STM and AFM, respectively (Figure S30, Supporting Information).

**Figure 6:**
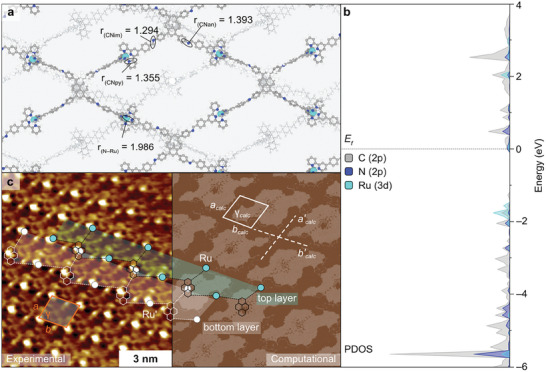
Modeling studies performed on the proposed the Ru‐pyrene MCOF structure. a) Optimised structure of the Ru‐pyrene MCOF model with relevant distances given in Å. The second layer of the 2D material is depicted in grey for clarity. b) Calculated projected density of states (PDOS) of the Ru‐pyrene MCOF structure in (a) with the contributions of the atomic orbitals highlighted in different colors. c) Experimental (left) and simulated (right) STM images of the Ru‐pyrene MCOF structure. The experimentally measured distances and angle (*a* = 1.7 ± 0.1 nm, *b* = 1.8 ± 0.1 nm, and *γ*  = 80 ± 3°) are shown in red. The interlayer distances obtained from the DFT‐optimized structure in (a) are shown in white (i.e.*, a_calc_* = 1.8 nm, *b_calc_*  =  1.8 nm, and *γ_calc_* = 60°). The experimental repeating units are superimposed in cyan (top layer) and white (bottom layer) for reference.

The proposed molecular model for the bilayer is presented in **Figure** **6**a, which shows a slip‐stacked AB type bilayer formed at the solution‐solid interface. Based on this model, we hypothesize that the lattice marked in Figure 5a corresponds to the [Ru(tpy)_2_]^2+^ moiety. This assumption is not unreasonable considering that the metal center and the orthogonal geometry of the two terpyridyl units are expected to enhance the STM contrast.^[^ ^52^ ^]^ The hypothesis is further supported by AFM height measurements of the films obtained after sonication of the solid Ru‐pyrene MCOF in heptanoic acid, followed by deposition on HOPG (without the presence of pre‐mixed monomers, in contrast to STM experiments) and drying of the sample, shown in Figure 5c. The bilayer morphology of the films is evident. Furthermore, the 2D crystallites deposited on the HOPG surface typically show a height difference of ca. 0.6 nm (Figure 5d). This value is in good agreement with the average distance of 0.54 nm between Ru and the C atom in the terpyridyl farthest away from the metal center in the DFT‐simulated model Ru‐pyrene MCOF (Figure 5b), which can be taken as the minimum expected interlayer spacing due to steric hindrance.

Periodic DFT calculations (see Supporting Information for details) were subsequently carried out to determine the atomic arrangement and corroborate the stacking of 2D layers in the Ru‐pyrene MCOF structure hypothesized by STM. The predicted lowest energy structure, including the relevant bond distances, is depicted in Figure 6a. This corresponds to an AB stacked structure, as suggested by STM and AFM measurements, where one of the layers is shifted by 12.594 and 7.245 Å along the *x* and *y* axes, respectively. This packing was found to be more stable than other AA and AB stackings (Figure S31, Supporting Information) by –0.22 and −0.15 eV, accordingly. Subsequently, the vibrational frequencies of the different C–N groups in the Ru‐pyrene framework were computed, leading to the identification of three types of C–N bonds, namely pyridinic (CN_py_), iminic (CN_im_) and anilinic (CN_an_). Further frequency calculations on the Ru‐pyrene 3 confirmed that the CN_im_ stretching of the MCOF overlaps with the terpyridine bands (Table S32, Supporting Information), in line with the experimental observations (Figure S12, Supporting Information), in the range between 1550 and 1700 cm^−1^.

We next sought to confirm that the bright spots in the experimental STM image (Figure 5a) correspond to the Ru atoms present in the framework. For this, we note that at negative potentials *V*, the energy of the Fermi level (*E_f_*) of the sample is raised by –*eV*. Thus, electrons in the sample can tunnel from the highest occupied states below *E_f_* into the unoccupied states of the tip. Moreover, two main features can enhance STM contrast, namely the presence of surface ledges, which increase the tunneling current by decreasing the distance between sample and tip,^[^ ^53^ ^]^ and a high local density of states below *E_f_*.^[^ ^54^ ^]^ For both reasons, we envisioned the [Ru(tpy)_2_]^2+^ moieties to provide a greater response in the STM measurements compared to the neighboring aromatic scaffold, as the stereochemistry of the terpyridyl ligands caused them to emerge from the flat framework imposed by the condensate benzene rings. In addition, the calculated projected density of states (PDOS) of the Ru‐pyrene MCOF model, shown in Figure 6b revealed that the first peaks below *E_f_* correspond to C and Ru atoms. Although their intensity is similar, the number of C atoms in the unit cell is much larger than Ru (512 vs 8 atoms), therefore, we expect the fraction of local density of states of Ru atoms in the occupied levels below *E_f_* to be significantly higher. This was confirmed by comparing the PDOS of a single Ru and C atoms within the Ru‐pyrene MCOF structure (Figure S31c, Supporting Information). Hence, we can conclude that the bright spots in the experimental STM image indeed correspond to the environment immediately surrounding the Ru atoms.

By using the above DFT model, we then simulated the STM profile and compared it to the experimental image, both depicted in Figure 6c. Both along the *a* and *b* directions, DFT calculations predict the intralayer spacing between adjacent Ru atoms to be of 3.9 nm (*a’_calc_* and *b’_calc_*), while the interlayer distance between Ru atoms is *a_calc_*  =  *b_calc_*  =  1.8 nm (Figure 6a). This latter value is in accordance with the STM experimental measurements for both *a* and *b* lengths, i.e., 1.7 ± 0.1 nm and 1.8 ± 0.1 nm, respectively, corroborating the AB‐stacked nature of the Ru‐pyrene MCOF. The angle between Ru atoms along the *a* and *b* directions was calculated to be *γ_calc_*  = 60°, in discrete agreement with the value of 80° ± 3° found experimentally. We note, however, that the value of *γ* strongly depends on the intralayer Ru–Ru distances *d_1_* and *d_2_* (Figure S33, Supporting Information). In light of this, we estimated that *d_1_* and *d_2_* values of 2.3 and 2.8 nm, respectively, would result in the experimental angle of *γ* = 80° while maintaining the *a_calc_* and *b_calc_* lattice vectors length of 1.8 nm. These *d_1_* and *d_2_* intralayer distances compare reasonably well with the DFT values of 2.1 and 3.2 nm, accordingly. Afterward, the distance between the uppermost C atom of a pyridine ligand and the C of the pyrene core in the subsequent layer was calculated to be 1.6 nm (Figure S34 and Table S35, Supporting Information), which matches well the thickness determined experimentally by AFM, ca. 1.5 nm (Figure 5d).

**Figure 7:**
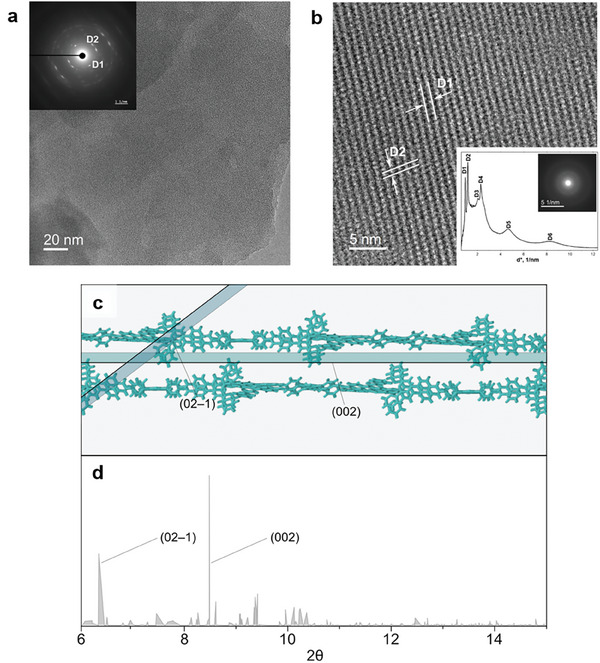
High resolution transmission electron microscopy (HR‐TEM) images of the Ru‐pyrene MCOF. a) Large area HR‐TEM image, inset shows a SAED pattern obtained from the Ru‐pyrene MCOF sample. b) Small area HR‐TEM image with defined lattice fringes. The inset describes the azimuthal averaged profile of the SAED pattern obtained from a large area of the Ru‐pyrene MCOF sample. c) Visual representation of the (02–1) and (002) Miller planes, cutting through the cell of the Ru‐pyrene MCOF framework reported in Figure 6a. d) Detailed zoom‐in view of the simulated PXRD pattern between 6° and 15° for the Ru‐pyrene MCOF model displayed in Figure 6a.

Subsequently, we set out to simulate the PXRD spectrum of the Ru‐pyrene MCOF (**Figure** **7**d). To accurately represent the spacing of the diffraction planes intersecting the *c*‐axis, the vacuum in the direction perpendicular to the 2D structure was removed, reproducing a uniform stacking of the A and B layers along the vertical direction (see Supporting Information for details). Coherently with the experimental findings and with the large dimensions of the unit cell, intense peaks were found at low 2θ values. Furthermore, a comparison between the computed and experimental PXRD spectra between 2–11 degrees (2θ), is presented in Table S36 and Figure S37 (Supporting Information). Although a good correlation is observed between experimental and simulated patterns, discrepancies can be attributed to long‐range stacking disorder within the well‐defined local structures and the inherent limitations of low‐angle PXRD acquisition.^[^ ^55^, ^56^ ^]^ By introducing slight stacking disorders in the computational models, we observed shifts of diffraction peak positions and changes in relative intensity (Table S38, Supporting Information), highlighting the challenges of correlating experimental and theoretical data in such layered systems.

HR‐TEM measurements were performed to provide further insights into the morphology, layered structure, and crystallinity of the Ru‐pyrene MCOF system at the nanoscale. The HR‐TEM images and corresponding selected‐area electron diffraction (SAED) patterns were recorded in low‐dose conditions to prevent the decomposition of the MCOF‐ordered structure. The contribution of amorphous layers was filtered in order to enhance the contrast from the crystalline phase in the images.^[^ ^57^ ^]^ The recorded images and SAED patterns confirmed that the framework consists of flake‐like crystallites, while dissimilar contrast on the image indicates a different number of layers, as shown in Figure 7. SAEDs taken from large areas showed polycrystalline diffraction (Figure 7b inset), while the d‐values obtained from these patterns (D1 = 10.50 Å, D2 = 8.60 Å, D3 = 4.90 Å, D4 = 4.40 Å, D5 = 2.15 Å, D6 = 1.20 Å) correspond well with the interplanar distances of ca. 10.39 and 8.43 Å, calculated from the experimental PXRD pattern (Table S16, Supporting Information). Individual crystallites showed single crystalline SAED patterns, although these are not perfect. The broadening of individual reflections and the presence of diffuse streaks indicate a planar disorder as evidenced by the SEM image shown in Figure 4a. This is most probably due to the presence of rotated or shifted 2D layers present within the bilayer structure, as suggested by DFT modeling and STM studies, making it even more challenging to obtain a detailed picture of the MCOF framework at the atomic level.

For this reason, we set out to further investigate the distances D1 and D2 highlighted by TEM on the calculated PXRD pattern reported in Figure 6d. To this aim, we explored the region of 2θ comprised between 6° and 15°, identifying the two most intense signals. The first one, which we relate to D1, appears at 6.28° and corresponds to the spacing between (02–1) Miller surfaces (d‐value ≃ 14.06 Å) passing through the Ru centers. The second one, assigned to D2, appears at 8.30° and relates to the spacing between (002) surfaces (d‐value ≃ 10.63 Å) cutting through the MCOF layers (Figure 7c). It is important to highlight that the discrepancy between the calculated and experimental values for D1 and D2 is significantly influenced by the interlayer distance. In particular, a reduction in this distance leads to a shortening of the *c*‐axis, which consequently reduces the spacing between the diffraction planes intersecting it.

Overall, the unique combination of STM, AFM, and HR‐TEM advanced microscopy techniques along with DFT studies confirmed the 2D nature of the Ru‐pyrene MCOF stacked as an AB shifted system with respect to the *x* and *y* axes. Despite the difficulties in fully characterizing this type of material at the atomic scale, the synergistic use of experimental and theoretical tools has successfully provided a complete atomic mapping of the Ru‐pyrene MCOF.

## 4 Conclusion and Future Outlook

In summary, in this work, we report the synthesis of a novel photoactive Schiff‐base MCOF linked through a ruthenium bis‐terpyridine metalloligand. The resulting porous ordered material has been characterized by a range of advanced microscopy techniques, including SEM, TEM, AFM, and STM, as well as TGA‐DSC, UV‐Vis, cyclic voltammetry, and periodic DFT calculations. Only by combining these experimental and computational techniques, we can confirm the semi‐planar 2D structure and provide unequivocally the atomic composition, as well as the unique layered framework produced upon linking the Ru metalloligand and a pyrene core. The Ru‐pyrene MCOF exhibits a well‐ordered 2D structure consisting of layers characterized by an AB shifted stacking, wherein the layers are separated by a shift of 12.594 Å along the *x* axis and 7.245 Å along the *y* axis. The polycrystalline nature of the MCOF is demonstrated by HR‐TEM, but controlling sample preparation with sufficient exfoliation allows for the isolation of few‐layer films. Importantly, this novel structure preserves the optical, thermal, and electrochemical properties of the Ru center intact while preventing the π‐stacking of the pyrene units, giving it potential application in thin‐film solar‐to‐energy and solar‐to‐chemical conversion. Further efforts to unravel the light‐driven charge transfer within the photoactive Ru(tpy)_2_ and pyrene units are ongoing, which will provide guidelines for the preparation of MCOFs in various optoelectronic and photocatalytic fields. Overall, this work expands the field of characterization of complex semi‐planar materials, in particular regarding the atomic‐level structure, to better gain insight into the potential application of such challenging systems.

## Conflict of Interest

The authors declare no conflict of interest.

## Supporting information



Supporting Information

## Data Availability

The data that support the findings of this study are available in the supplementary material of this article.
