# Supplementary material for: Unravelling the Atomic Structure of a Metal‐Covalent Organic Framework Assembled from Ruthenium Metalloligands
Source: Adv Mater. 2025 Feb 19;37(13):2502155. doi: 10.1002/adma.202502155 (PMC11962698; doi:10.1002/adma.202502155)
Supplement: Supplementary file 1 — Supporting Information [file ADMA-37-2502155-s001.docx]

Supporting Information

Unravelling the atomic structure of a metal-covalent organic framework assembled from ruthenium metalloligands

Seán Hennessey,^[a]^ Roberto González-Gómez,*^[a]^ Nicolás Arisnabarreta,^[b]±^ Anna Ciotti,^[c]±^ Jing Hou,^[d]^ Nadezda V. Tarakina,^[d]^ Andrey Bezrukov,^[e]^ Kunal S. Mali,^[b]^ Michael Zaworotko,^[e]^ Steven De Feyter,^[b]^ Max García-Melchor,*^[c]^ Pau Farràs*^[a]^

[a] Dr. Seán Hennessey, Dr. Roberto González-Gómez, Dr. Pau Farràs

School of Biological and Chemical Sciences, Energy Research Centre, Ryan Institute, University of Galway

University Road, Galway, Ireland, H91 TK33

E-mail: [pau.farras@universityofgalway.ie](mailto:pau.farras@universityofgalway.ie); roberto.gonzalez@universityofgalway.ie

[b] Dr. Nicolas Arisnabarreta, Dr. Kunal S. Mali, Prof. Steven De Feyter
 Division of Molecular Imaging and Photonics, Department of Chemistry, KU Leuven

Celestijnenlaan 200F, 3001 Leuven, Belgium

[c] Anna Ciotti, Prof. Max García-Melchor

School of Chemistry, CRANN and AMBER Research Centres, Trinity College Dublin

College Green, Dublin 2, Ireland, D02 PN40

[d] Dr. Jing Hou, Dr. Nadezda V. Tarakina

Max-Planck-Institut für Kolloidund Grenzflächenforschung

Am Mühlenberg 1, 14476 Potsdam, Germany

[e] Dr. Andrey Bezrukov, Prof. Michael Zaworotko

Bernal Institute, University of Limerick

Limerick, Ireland, V94 T9PX

[f] Prof. Max García-Melchor

Center for Cooperative Research on Alternative Energy (CIC EnergiGUNE), Basque Research and Technology Alliance (BRTA), Alava Technology Park, Albert Einstein 48, 01510 Vitoria- Gasteiz, Spain.

[g] Prof. Max García-Melchor

IKERBASQUE, Basque Foundation for Science, Plaza de Euskadi 5, 48009 Bilbao, Spain.

E-mail: maxgarcia@cicenergigune.com

± These authors contributed equally

**Table of Contents**

1. Experimental Procedures 2

1.1. General Experimental Techniques 2

1.2. Chemicals And Solvents 2

1.3. Nuclear Magnetic Resonance (NMR) Spectroscopy 3

1.4. Infrared (IR) Spectroscopy 4

1.5. Mass Spectrometry (MS) 4

1.6. Powder X-Ray Diffraction (PXRD) 4

1.7. Thermal Gravitational Analysis-Differential Scanning Calorimetry (TGA-DSC) 4

1.8. Inductively Coupled Plasma-Optical Emission Spectrometry (ICP-OES) 5

1.9. Electrochemistry 5

1.10. Photophysical Measurements 5

1.11. Microscopy 5

1.12. Brunauer–Emmett–Teller (BET) Analysis 7

2.1. Synthetic Procedures 9

2.1. Synthesis of 4-(diethoxymethyl)-phenyl-4-[2,2';6',2''-terpyridine] (1) 9

2.2. Synthesis of 4-[2,2';6',2'']-terpyridin-4-benzaldehyde (2) 10

2.3. Synthesis of [Ru(1)_2_](2PF_6_) (3) 10

2.4. Synthesis of Ru-Pyrene MCOF 11

3. Characterisations 12

3.1. 4-(Diethoxymethyl)-phenyl-4-[2,2’;6’,2’’-terpyridine] (1) 12

3.2. 4-[2,2';6',2'']-Terpyridin-4-benzaldehyde (2) 13

3.3 [Ru(1)_2_](2PF_6_) (3) 15

3.4. Ru-Pyrene MCOF 17

References 33

Author Contributions 36

1. Experimental Procedures

1.1. General Experimental Techniques

The newly synthesized organic molecules are named according to IUPAC naming conventions. The numbering in the carbon chain is based on the position of the carbon atom. Thin layer chromatography (TLC) was performed on aluminium-coated plates with either Merck TLC silica gel 60 F254 plates or aluminium oxide TLC plates. Visualization was achieved using a UV lamp (254 nm) or charring with KMnO_4_. Flash chromatography was carried out with silica gel (pore size 60 Å, 230–400 mesh, and particle size 40–63 µm) or aluminium oxide (Neutral, Brockmann I), using a stepwise solvent polarity gradient correlated with TLC mobility. Individual solvent systems are reported in the characterization section of each compound.

1.2. Chemicals and Solvents

| **Chemical Reagent** | **Supplier** | **Reported Purity (%)** |
| --- | --- | --- |
| 2-Acetylpyridine | Fluorochem | 98.0 |
| 4-(4,4,5,5-Tetramethyl-1,3,2-dioxaborolan-2-yl)aniline | Fluorochem | 99.0 |
| 1,3,6,8-Tetrabromopyrene | TCI | > 98.0 |
| Argon | BOC | 99.8 |
| Ammonium hexafluorophosphate | Fluorochem |  |
| 4-(Diethoxymethyl)benzaldehyde | Fluorochem | 95.0 |
| Ammonium hydroxide | Sigma-Aldrich | 35.0 % (v/v) |
| Hydrogen peroxide | Fluka | 35.0 % (v/v) |
| Magnesium sulfate | Alfa Aesar | 99.5 |
| Mesitylene | Fluorochem | 99.0 |
| Nafion | Alfa Aesar | 5.0 % (w/w) |
| Poly(methyl methacrylate) (Mw ~120,000) | Sigma-Aldrich | > 98.0 |
| Potassium hydroxide | Sigma-Aldrich | ≥ 85.0 |
| Ruthenium chloride | TCI | 99.9 |
| Silica gel | Sigma-Aldrich | N/A |
| Tetrabutylammonium hexafluorophosphate (Electrochemical grade) | Sigma-Aldrich | 99.9 |
| Tetrakis(triphenylphosphine)palladium(0) | Sigma-Aldrich | 99.0 |
| **Solvent** | **Supplier** | **Reported Purity (%)** |
| 1,4-Dioxane | Sigma-Aldrich | 99.8 |
| Acetic acid | Sigma-Aldrich | ≥ 99.0 |
| Acetone | Fischer Scientific | > 99.0 |
| Acetonitrile | Sigma-Aldrich (HPLC Plus) | ≥ 99.9 |
| Cyclohexane | Sigma-Aldrich | > 99.0 |
| Diethyl ether | Sigma-Aldrich | > 99.5 |
| Dimethyl sulfoxide | Sigma-Aldrich | > 99.8 |
| Ethanol | Fischer Scientific | > 99.0 |
| Ethyl acetate | Fischer Scientific | ≥ 99.0 |
| Methanol | Sigma-Aldrich (HPLC Grade) | ≥ 99.9 |
| Tetrahydrofuran | Sigma-Aldrich (CHROMASOLV® Plus) | ≥ 99.9 |

1.3. Nuclear Magnetic Resonance (NMR) Spectroscopy

All NMR data are reported in the following order: chemical shift (δ) in ppm with multiplicities indicated as s (singlet), d (doublet), dd (doublet of doublets), t (triplet), td (triplet of dublets), q (quartet), m (multiplet) and coupling constants (*J*) given in hertz (Hz). Chemical shifts are reported relative to internal standard Si(CH_3_)_4_ in CDCl_3_ for ^1^H-NMR and CDCl_3_ for ^13^C-NMR. NMR data for known compounds were in good agreement with previously published data and are referenced throughout this work.

1.3.1. Liquid NMR

Liquid samples were carried out at the “Galway NMR Services Centre” in the School of Biological and Chemical Sciences, University of Galway. Spectra were recorded using a Varian VNMRS 500 MHz 54mm AR Spectrometer. ^1^H-NMR spectra were recorded at 500 MHz using Bruker spectrometers and processed with Bruker Topspin software calibrated against solvent peaks according to published values. ^13^C-NMR spectra were recorded at 126 MHz as indicated using Bruker spectrometers and were processed in the same way.

1.3.2. Solid-State NMR

Solid-state NMRs were carried out at the “NMR Spectroscopy Facility” in the School of Chemistry, Trinity College Dublin. Spectra were recorded using a Bruker Avance HD 400 NMR equipped with a 5.0 mm BBFO probe for proton and multinuclear detection, an automatic sample changer and a 3.2 mm H/X CP-MAS probe for solid-state NMR.

1.4. Infrared (IR) Spectroscopy

IR spectra (4000–650 cm^−1^) were recorded using a PerkinElmer 16PC FT-IR spectrometer with a KBr reference.

1.5. Mass Spectrometry (MS)

High-resolution mass spectra were measured in positive or negative mode as indicated using a Waters LCT Mass Spectrometry instrument.

1.6. Powder X-Ray Diffraction (PXRD)

PXRD data for PyTTA and **3** were recorded in the “Centre for Crystallography” at the University of Galway. Data was collected using an Inex Equinox 6000 diffractometer. Real-time measurements were performed in θ/2θ mode with an attached high-precision Eulerian cradle. The MCOF sample was measured at the “X-Ray Crystallography Facility” at Trinity College Dublin. The sample was packed into a polyimide tube (ID 0.0249“), sealed with wax and mounted on a MiTeGen goniometer base. Data was collected at room temperature on a Bruker APEX DUO with a Cu Iμs microfocus source and an APEX 2 detector, using four consecutive 2θ runs of 600s each. The data were combined and integrated using APEX 3 Debye1 ring plug-in.

1.6.1. Small-Angle X-Ray Scattering (SAXS)

SAXS data for Ru-pyrene MCOF was recorded in the "Warwick Scientific Services” specialised centre at the University of Warwick. Measurements were acquired in a Xeuss 2.0 equipped with a Cu Kα microfocus X-ray source. Data was recorded between 0.3 and 15.6° 2θ. Two measurement positions were used (detector translated in z) to increase the angular range. The two images were combined and the output scattering was integrated to give 1d intensity vs q data. The powder sample was mounted between two Scotch tape windows and an empty cell was measured for background correction. The scattering from the empty cell has been subtracted from the measured data of the sample. The final output data shows weak peaks at 5.2 and 7° 2θ attributed to differences in the background scattering between instruments and different signal-to-noise values.

1.7. Thermal Gravitational Analysis-Differential Scanning Calorimetry (TGA-DSC)

TGA-DSC experiments were performed on an STA625 thermal analyzer from Rheometric Scientific (Piscataway, New Jersey). The samples were measured from 20 to 600 °C with a heating rate of 10 °C min^-1^. The measurements were performed using open aluminium crucibles, nitrogen was purged in ambient mode, and calibration was performed using an indium standard.

1.8. Inductively Coupled Plasma-Optical Emission Spectrometry (ICP-OES)

Metal concentration analysis was performed in triplicate with an inductively coupled plasma-optical emission spectrophotometer (ICP-OES 5110). The operational conditions of the ICP-OES spectrometer were RF power: 1200 W, argon plasma flow rate: 12.0 L min^−1^, auxiliary argon flow rate: 1.0 L min^−1^, and nebulizer argon flow rate: 0.7 L min^−1^. Ruthenium content was read on radial mode at 267.876 nm.

1.9. Electrochemistry

Electrochemistry measurements for cyclic voltammetry (CV) were performed in a PalmSense-3 potentiostat at room temperature with N_2_-bubbled dry CH_3_CN and a 0.1 M ^n^Bu₄PF₆ supporting electrolyte, using a scan rate of 0.1 V s^-1^. A glassy carbon (GC) as the working electrode, platinum as the counter electrode, and a standard saturated calomel electrode (SCE) as the reference, were used for the three-electrode set-up. Ru-pyrene MCOF measurements were performed by its immobilization on the GC surface *via* a Nafion/EtOH suspension, achieved by sonicating for 30 min before drop casting onto the electrode surface.

1.10. Photophysical Measurements

1.10.1. Ultraviolet-Visible (UV-Vis) Spectroscopy

UV-Vis measurements were carried out on a Cary 5000 UV-Vis-NIR spectrometer (200-2500 nm range) with a deuterium UV lamp light source using R928PTM (UV-Vis), thermoelectrically cooled PbS (NIR) or polytetrafluoroethylene (diffuse reflectance spectroscopy (DRS)) detectors using a xenon lamp.

1.10.2. Fluorescence Spectroscopy

Emission measurements were recorded on a Cary Eclipse fluorescence spectrophotometer with a multicell holder for liquids and a diffuse reflectance accessory (DRA) for solids.

1.11. Microscopy

1.11.1. Atomic Force Microscopy (AFM)

AFM was performed on a Veeco Dimension 3100 AFM. Scout 350R general-purpose silicon probes by Nunano were used for analysis. For measurements, samples were suspended in 1 mL of ethanol and sonicated for 10 min at room temperature. 2 µL of the suspension was drop cast on mica discs (grade V1 mica, 0.21 mm thickness, 9.9 mm diameter, Agar Scientific) ­*via* spin coating and dried at room temperature for 24 h before measurements.

MCOF powder was firstly dispersed in heptanoic acid and sonicated for 15 min, reaching a suspension. Afterwards, it was carefully dropcasted onto freshly cleaved highly oriented pyrolytic graphite (HOPG) and dried on a hotplate at 50 °C. Once dried, AFM measurements were carried out using a Cypher ES (Asylum research) system using tapping mode in the air at room temperature. OMCL-AC240TS probes (spring constant ~ 2 N/m) with a resonance frequency of around 70 KHz were employed.

1.11.2. Scanning Tunneling Microscopy (STM)

STM experiments were performed using a Molecular Imaging (Agilent Technologies) or PicoLE (Keysight) STM operating in constant-current mode at room temperature. The tips of Pt/Ir wire (80/20, diameter 0.25 mm, Advent Research Materials) were mechanically cut. STM parameters, V_bias_ and I_set_, are indicated in each figure corresponding to the applied sample potential and current, respectively. High-resolution STM images were calibrated (when indicated) using the underneath graphite lattice, using Scanning Probe Image Processor (SPIP, Image Metrology) software. Prior to every experiment, the HOPG substrate (grade ZYB, Momentive Performance Material Quartz Inc., Strongsville, OH, USA) was freshly cleaved using Scotch tape. Solutions were prepared using as-received n-heptanoic acid (HA, Merck). A pre-mixture of monomers dissolved in HA was dropcasted onto HOPG, and a pinch of the MCOF was also incorporated into the surface prior to STM scanning.

1.11.3. Scanning Electron Microscopy-Energy Dispersive X-ray Analysis (SEM-EDX)

SEM-EDX measurements were performed at the “Bernal Institute” in the University of Limerick. The measurements were conducted using a Tescan Maia3 instrument operating at 5 or 20 kV. Samples were dispersed in absolute ethanol *via* 30–60 min sonication. The resulting mixtures were then dropcasted and dried on clean silicon wafers (5 x 5 mm). The final samples were coated with gold before recording the SEM measurements.

1.11.4. Transmission Electron Microscopy (TEM)

TEM measurements were carried out on a Hitachi H7500 electron microscope. All images were taken at 100 kV at room temperature and shown in their raw data form. Non-exfoliated samples were dispersed in ethanol under brief sonication before being drop-casted on 200 mesh copper grids with Formvar/Carbon membrane coatings. Exfoliated samples were sonicated in ethanol at 50 °C for 6 h before deposition using the same method as the non-exfoliated sample.

1.11.5. High-Resolution Transmission Electron Microscopy (HR-TEM)

For HR-TEM, a suspension of the non-exfoliated sample was sonicated in ethanol for 10 min, then drop-casted onto a Cu grid with a lacey carbon support and dried for 15 min. TEM studies were performed using a double Cs corrected JEOL JEM-ARM200F (S)TEM operated at 80 kV and equipped with a cold-field emission gun. Images were recorded using a OneView Gatan camera in low-dose conditions with the electron flux optimized to 12 e/Å^2^s. HR-TEM images were filtered using the “HR-TEM Filter” plugin for Digital Micrograph Suite developed by D. Mitchell based on.^[1]^ In this plugin, a Wiener filter and an average background subtraction filter are applied to enhance the crystalline contrast on the images in the presence of an amorphous layer.

1.12. Brunauer–Emmett–Teller (BET) Analysis

Gas adsorption measurements were carried out at the Bernal Institute, University of Limerick. For gas sorption experiments, ultrahigh-purity gases were used as received from BOC Gases: research-grade He, CO_2_ (99.995%). Adsorption experiments at pressure up to 1 bar for CO_2_ were performed on a Micromeritics 3Star surface characterization analyzer. Before sorption measurements, 50 mg of the sample was activated on a SmartVacPrep™ using a dynamic vacuum for 12 h. The low-temperature experiments at 195 K were controlled using a 4 L Dewar filled with dry ice/acetone. BET surface areas were determined from the CO_2_ adsorption isotherm at 195 K using the Micromeritics Microactive software.

**1.13. Computational Modelling**

Periodic density functional theory (DFT) calculations were performed with the Vienna ab initio simulation package (VASP, version 6.3.1),^[2]^ adopting the Perdew-Burke-Erzernhof exchange-correlation functional^[3]^ and Grimme’s D3 dispersion corrections to account for van der Waals interactions.^[4]^ Core electrons were described through projector-augmented wave pseudopotentials,^[5]^ while valence electrons were described by plane waves with a kinetic energy cut-off of 500 eV. The occupancies of the electronic energy levels were described with a Gaussian smearing of width 0.05 eV, and an energy threshold of 10^–6^ eV was adopted for the convergence of the electronic steps. After each electronic loop, the initial and final electron densities were combined to provide a guess of the input electron density for the next iteration according to the linear mixing approach within Johnson’s modification of the Broyden scheme,^[6]^ using parameters of 0.2 and 0.0001, and 0.8 and 0.0001 for the magnetization density.

The Ru-pyrene MCOF model structures were built with the software Atomic Simulation Environment (ASE).^[7]^ In particular, AA stacking was considered, where the atoms of all layers are perfectly superimposed on one another (Figure S30a). Additionally, two different AB stackings were proposed. In the first one, a shift of 15.130 Å and 3.381 Å was applied along the *x* and *y* axes (Figure S30b), respectively, whereas in the second one, a shift of 12.594 and 7.245 Å was applied along the *x* and *y* axes (Figure 6a), accordingly. In all stackings, an interplanar distance of 5 Å was initially set between layers along the *z* direction (perpendicular to the MCOF plane) to allow the establishment of van der Waals interactions between layers and a vacuum of at least 15 Å was imposed between periodic images. The length of the *x* and *y* axes was 6.37 and 4.26 nm, respectively, estimated as the distance between two corresponding atoms belonging to neighbouring images in the model structure of the Ru-pyrene MCOF layer before optimization.

Due to the considerable size of the modelled Ru-pyrene MCOF (912 atoms), structural relaxations were carried out at the 𝛤-point using the conjugate-gradient algorithm^[8]^ and a step-size of 0.1 Å. These were performed by allowing the lattice vectors, cell shape, and atomic positions to relax until the forces acting on all atoms were ≤ 0.01 Å eV^–1^. The projected density of states (PDOS) was determined via a single point calculation using a denser 𝛤-centered 3×3×1 k-point mesh with the tetrahedron smearing method with Blöchl corrections^[9]^ using a width of 0.05 eV. The PDOS was plotted with the Sumo software, version 2.3.5.^[10]^

The C–N vibrational frequencies were computed through the finite difference method^[11]^ with a step size of 0.02 Å, allowing only the relevant C and N atoms to vibrate while keeping all the other atoms in the Ru-pyrene MCOF fixed in their optimized positions.

To confirm the overlap between the IR bands of the imines in the Ru-pyrene MCOF and the terpyridines of complex **3**, the latter was optimized, with an energy convergence criterion of 10^–4^ eV for both the electronic and ionic steps, constraining the position of Ru while allowing the ligand atoms to relax. In this calculation, the ethoxy functional groups were substituted with H atoms as they are not expected to show any vibrational modes in the region of interest (1500–1650 cm^–1^). A frequency calculation was then run, lowering the precision and switching the algorithm for the orbitals optimization to the preconditioned residuum-minimization to reduce the computational cost.^[12]^ This led to the identification of 14 peaks within the region of interest, summarized in Table S31, corresponding to the “breathing” of complex **3**.

The simulated STM image provided in Figure 6c was generated with the STM-2DScan code in constant current mode.^[13]^ To describe the crystal packing of the Ru-pyrene MCOF for the computation of the X-ray diffraction spectrum, the vacuum was removed and the minimum interlayer spacing was adjusted to match the value found in the previously optimized structures (*ca.*5.0 Å). The A-B interlayer distances for seven distinct c-axis lengths were computed, details of which are summarized in Table S34. These calculations were pivotal in selecting an optimal *c*-axis length of 21.44 Å, which provided the most consistent spacing between the A and B layers. The X-ray diffraction spectrum was subsequently simulated with the Visualization for Electronic and Structural Analysis (VESTA)^[14]^ software, imposing a single wavelength scan with λ = 1.541 Å. The peaks of intensity exceeding 5.0 % are presented in Table S35 and compared to the experimental values from Table S16.

2.1. Synthetic Procedures

All reactions involving air-sensitive or moisture-sensitive reagents were performed under an argon atmosphere. The used glass devices were heated under a vacuum with a heat gun (400 °C) prior to use. Solid chemical reagents were added under argon counterflow and liquid reagents via disposable syringes and needles. Paraffin oil baths were used as heat baths and mixtures of ice/water for low temperatures. The temperature was set and controlled *via* an adjustable contact thermometer. Ru(DMSO)_4_Cl_2_^[15]^ and PyTTA^[16]^ were synthesised according to previously reported literature procedures.

2.1. Synthesis of 4-(diethoxymethyl)-phenyl-4-[2,2';6',2''-terpyridine] (1)

A literature procedure was used as inspiration for this synthesis.^[17]^ 4-(diethoxymethyl)benzaldehyde (0.500 g, 2.40 mmol) was added to a stirring solution of 2-acetylpyridine (0.6 mL, 0.633 g, 5.35 mmol), NH_4_OH (2.5 mL, 35.0 % aqueous solution) in EtOH (25.0 mL). KOH (0.300 g, 5.35 mmol) in 8.0 mL of H_2_O was added dropwise over 20 min. The mixture was left stirring, at room temperature, for 24 h until a green, oily precipitate was formed. The precipitate was collected by filtration and purified by column chromatography on silica gel (EtOAc:cyclohexane, 5:95 → 50:50) to yield **1** as an analytically pure, bright yellow powder (520 mg, 42.6 %). **^1^H-NMR** (500 MHz, CDCl_3_) δ [ppm]: 8.76–8.71 (m, 4H), 8.67 (d, *J* = 8.0 Hz, 2H), 7.93–7.86 (m, 4H), 7.62 (d, *J* = 8.1 Hz, 2H), 7.35 (dd, 2H), 5.59 (s, 1H), 3.65 (q, *J* = 6.9 Hz, 4H), 1.27 (t, *J* = 7.1 Hz, 6H). **^13^C-NMR** (126 MHz, CDCl_3_) δ [ppm]: 156.2, 155.9, 150.0, 149.1, 139.9, 138.5, 136.8, 127.3, 127.2, 123.8, 121.3, 118.9, 101.2, 61.0, 15.2. **LC-MS** m/z 412.20 [M+H]^+^.

2.2. Synthesis of 4-[2,2';6',2'']-terpyridin-4-benzaldehyde (2)

To a 50 mL RBF was added 4-(diethoxymethyl)-phenyl-4-[2,2';6',2''-terpyridine] (0.200 g, 0.49 mmol), which was dissolved in 15 mL of THF and cooled in an ice bath. 2 mL of conc. HCl was added dropwise, and the yellow solution was warmed to room temperature and refluxed for 3 h. The bright orange mixture was then cooled to 0 °C and neutralized by the dropwise addition of a saturated solution of K_2_CO_3_. The solvent was reduced, and the now-white solution was extracted with EtOAc (20 mL) and washed with H_2_O (2 x 10 mL) and brine (1 x 10 mL). The pale white solution isolated was dried over MgSO_4_, filtered and dried to give **2** as an analytically pure white powder (0.160 g, 97.0 %). **^1^H-NMR** (500 MHz, CDCl_3_) δ [ppm]: 10.10 (s, 1H), 8.73 (d, *J* = 4.0 Hz, 2H), 8.68 (d, *J* = 7.9 Hz, 2H), 8.04 (q, *J* = 8.2 Hz, 4H), 7.89 (td, *J* = 8.2, 1.9 Hz, 2H), 7.40–7.34 (m, 3H). **^13^C-NMR** (126 MHz, CDCl_3_) δ [ppm]: 191.8, 156.22, 155.8, 150.0, 149.1, 149.1, 148.8, 144.4, 136.9, 136.4, 130.2, 130.1, 128.0, 127.6, 124.0, 121.3, 119.0. **LC-MS**: m/z 338.13 [M+H]^+^.

2.3. Synthesis of [Ru(1)_2_](2PF_6_) (3)

A 25 mL Schlenk flask was charged with **1** (0.100 g, 0.24 mmol) and Ru(DMSO)_4_Cl_2_ (0.056 g, 0.12 mmol). The reaction flask was kept in the dark and placed under an argon atmosphere. A degassed ethanol/water (90:10, 10 mL) solution was added *via* a degassed syringe, and the reaction was left at reflux for 3 h. After cooling, the solvent was reduced and the red/orange solid isolated was dissolved in H­_2_O/EtOH (50:10). Dropwise addition of a saturated solution of NH_4_PF_6_ resulted in a bright red precipitate. Isolation *via* filtration, and recrystallization with a CH_3_CN/Et_2_O mixture resulted in **3** as a bright red powder (0.082 g, 58.0 %). **^1^H-NMR** (500 MHz, (CD_3_)_2_CO) δ [ppm]: 9.44 (s, 4H), 9.07 (d, *J* = 8.0 Hz, 4H), 8.36 (d, *J* = 8.0 Hz, 4H), 8.09 (s, 4H), 7.81 (d, *J* = 5.6 Hz, 8H), 7.34 (s, 4H), 5.71 (s, 2H), 3.73–3.63 (m, 8H), 1.27 (t, *J* = 7.0 Hz, 12H). **^13^C-NMR** (126-MHz, (CD_3_)_2_CO) δ [ppm]: 158.69, 156.34, 155.93, 155.90, 152.75, 149.90, 149.44, 148.22, 142.23, 141.04, 138.3, 138.3, 137.1, 136.6, 127.9, 127.9, 127.7, 127.6, 126.8, 124.9, 124.3, 121.6, 121.0, 118.4, 101.0, 61.0, 29.9, 14.8. **LC-MS** m/z 462.89 [M/2 – 2PF_6_ + H]^+^.

2.4. Synthesis of Ru-pyrene MCOF

PyTTA (0.011 g, 0.02 mmol) and [Ru(**1**)_2_](2PF_6_) (**3**) (0.049 g, 0.04 mmol) linkers were placed in a Pyrex tube (10 mL), followed by adding a solution of mesitylene/dioxane/6 M AcOH (5/5/1 by vol.; 2 mL). The mixture was sonicated for 10 min and then degassed by bubbling with N_2_ for 15 min. The reaction was heated at 115 °C for 5 d yielding a dark red precipitate, which was isolated by filtration. The wet sample was then thoroughly washed with THF (30 mL), acetonitrile (30 mL) and methanol (30 mL), after which the powder was taken up in 10 mL of methanol and sonicated for 3 h at 50 °C. The powder was isolated by filtration and dried under a vacuum overnight to give the MCOF as a dark red powder.

3. Characterisations

3.1. 4-(diethoxymethyl)-phenyl-4-[2,2’;6’,2’’-terpyridine] (1)


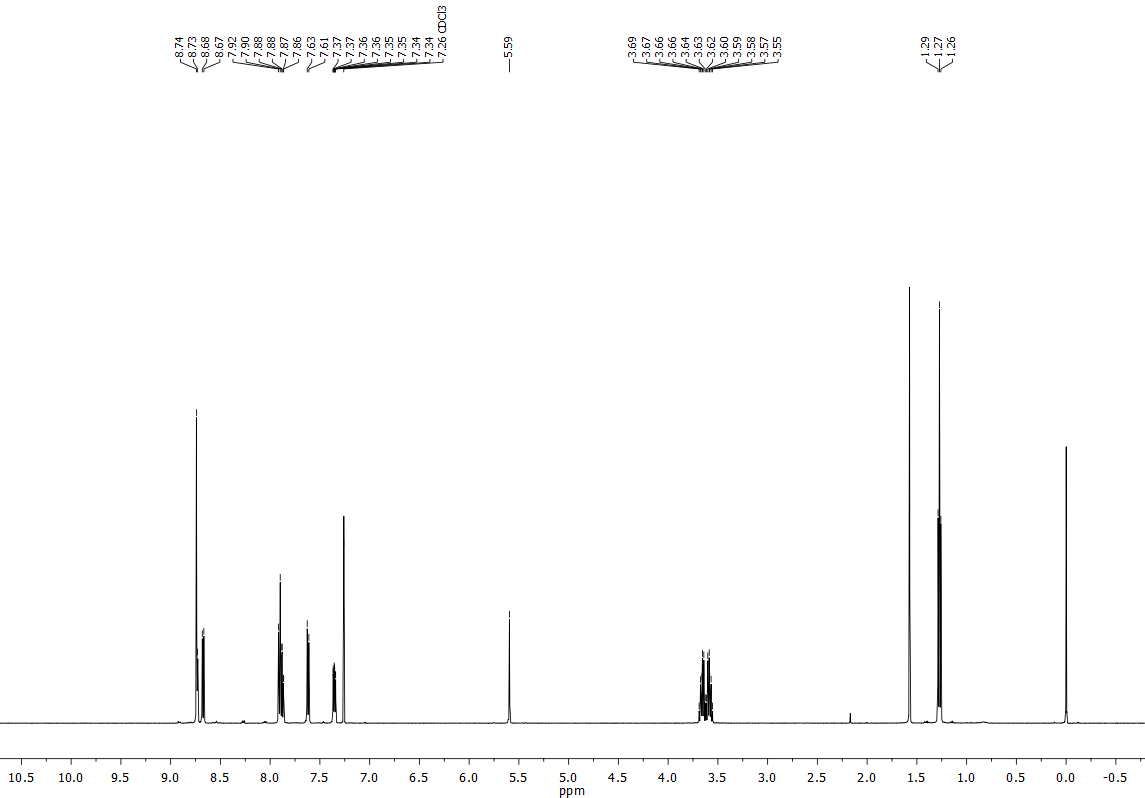


**Figure S1.** ^1^H-NMR (500 MHz, CDCl_3_) of 4-(diethoxymethyl)-phenyl-4-[2,2’;6’,2’’-terpyridine] (**1**).


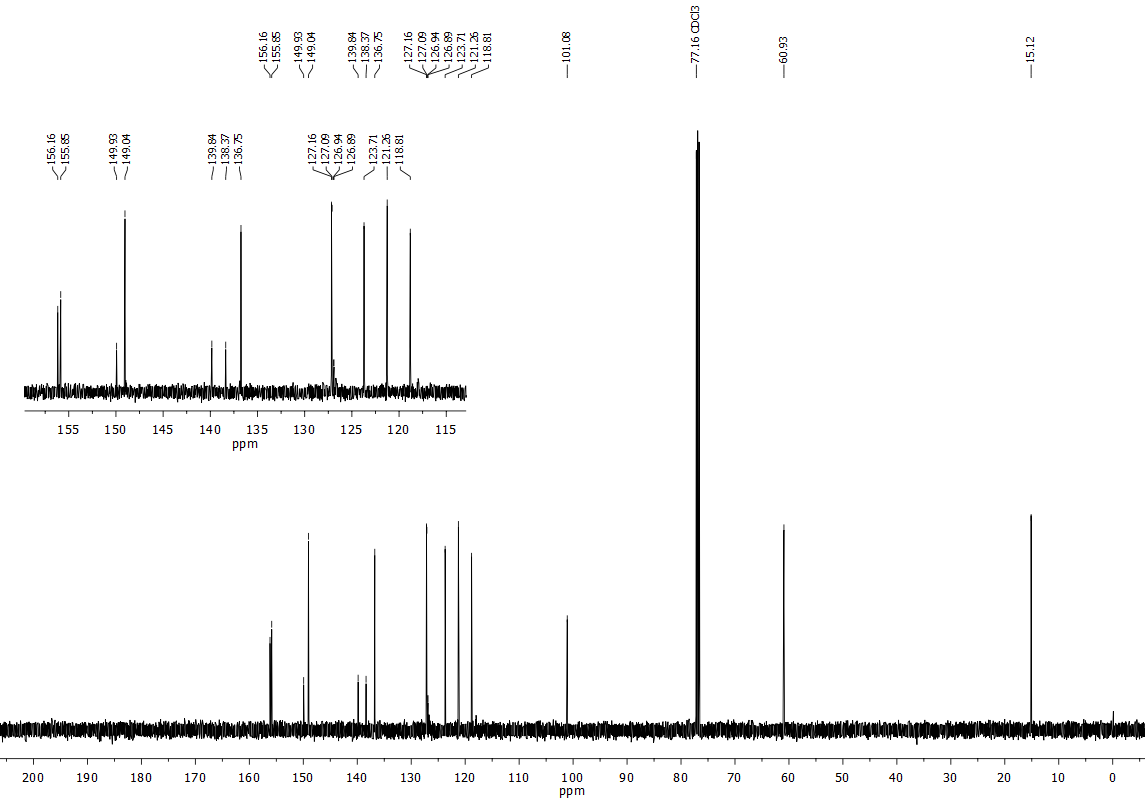


**Figure S2.** ^13^C-NMR (126 MHz, CDCl_3_) of 4-(diethoxymethyl)-phenyl-4-[2,2’;6’,2’’-terpyridine] (**1**).


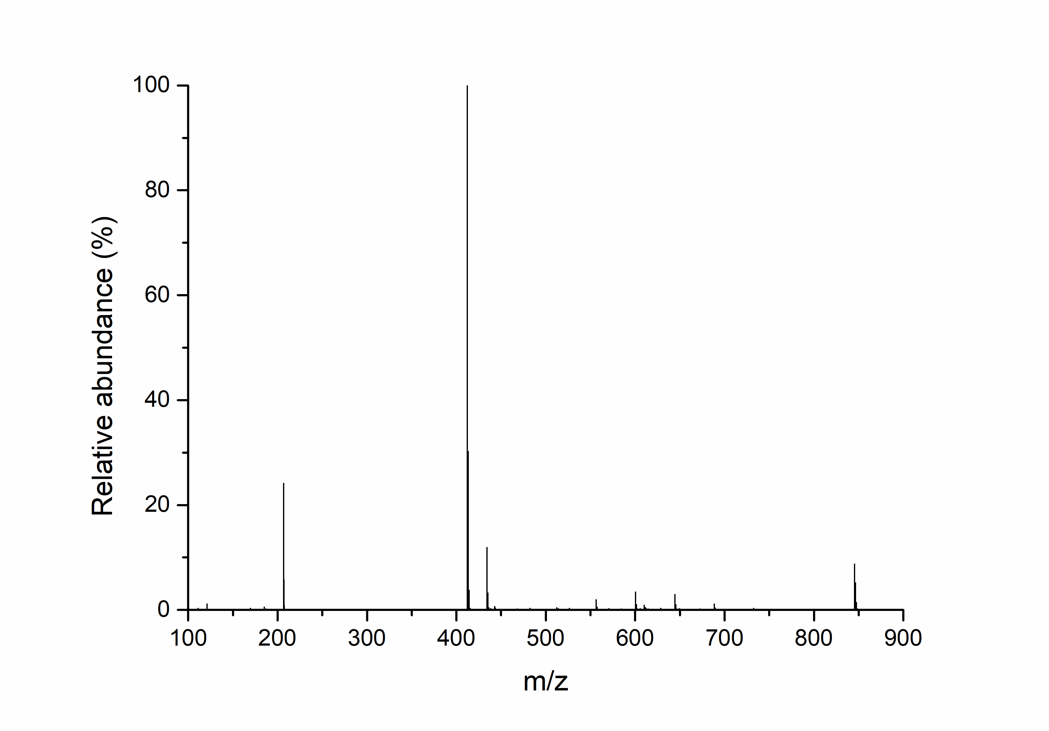


**Figure S3.** Positive mode ESI-MS of 4-(diethoxymethyl)-phenyl-4-[2,2’;6’,2’’-terpyridine] (**1**). [M+H]^+^: 412.20.

3.2. 4-[2,2';6',2'']-terpyridin-4-benzaldehyde (2)


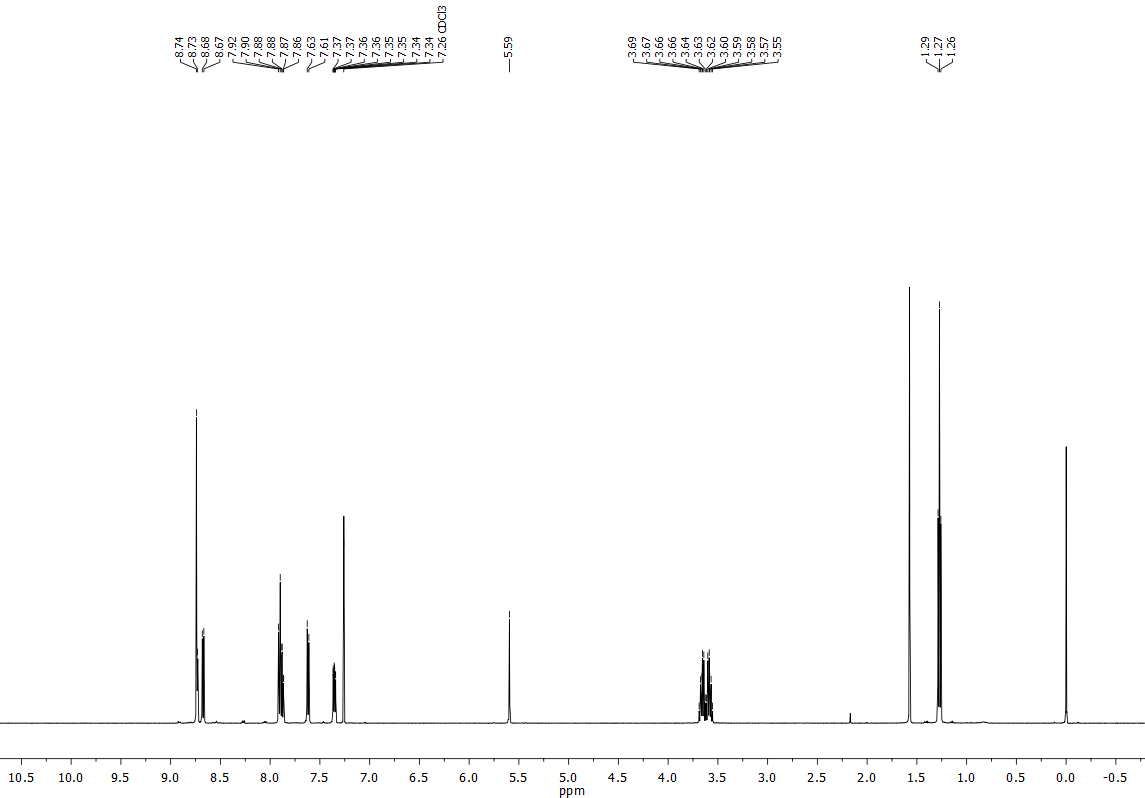


**Figure S4.** ^1^H-NMR (500 MHz, CDCl_3_) of 4-[2,2';6',2'']-terpyridin-4-benzaldehyde (**2**).


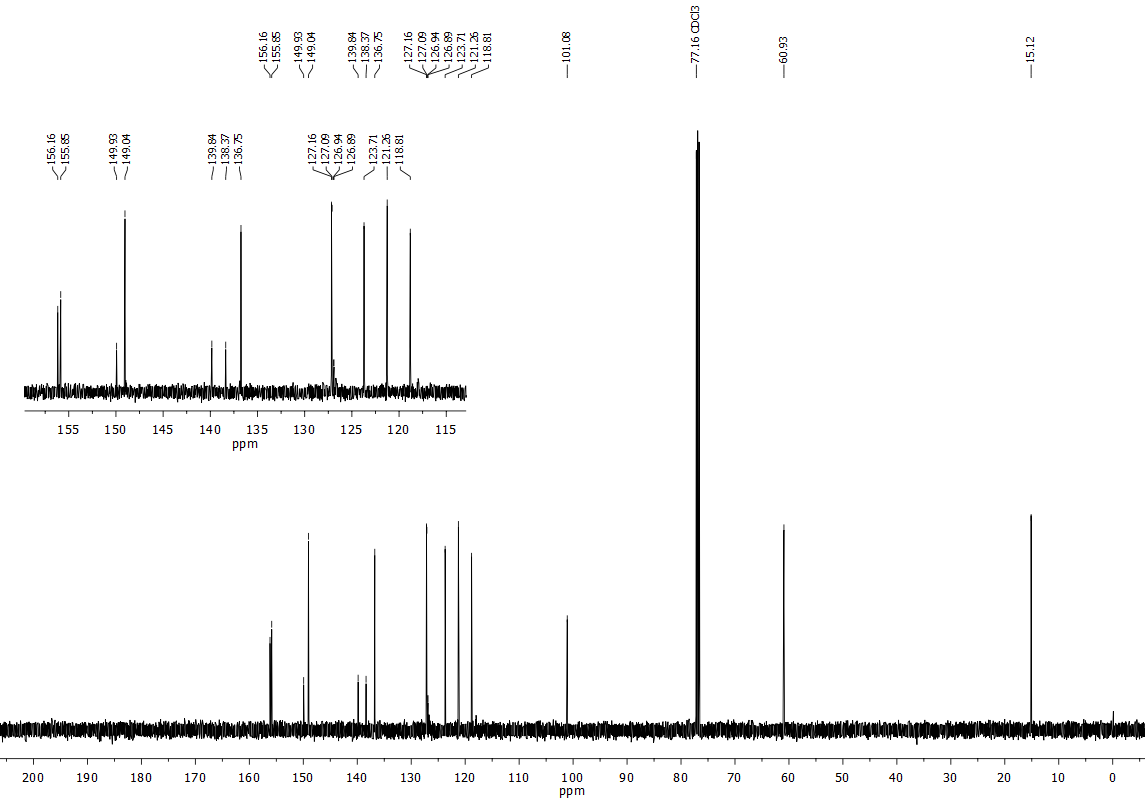


**Figure S5.** ^13^C-NMR (126 MHz, CDCl_3_) of 4-[2,2';6',2'']-terpyridin-4-benzaldehyde (**2**).


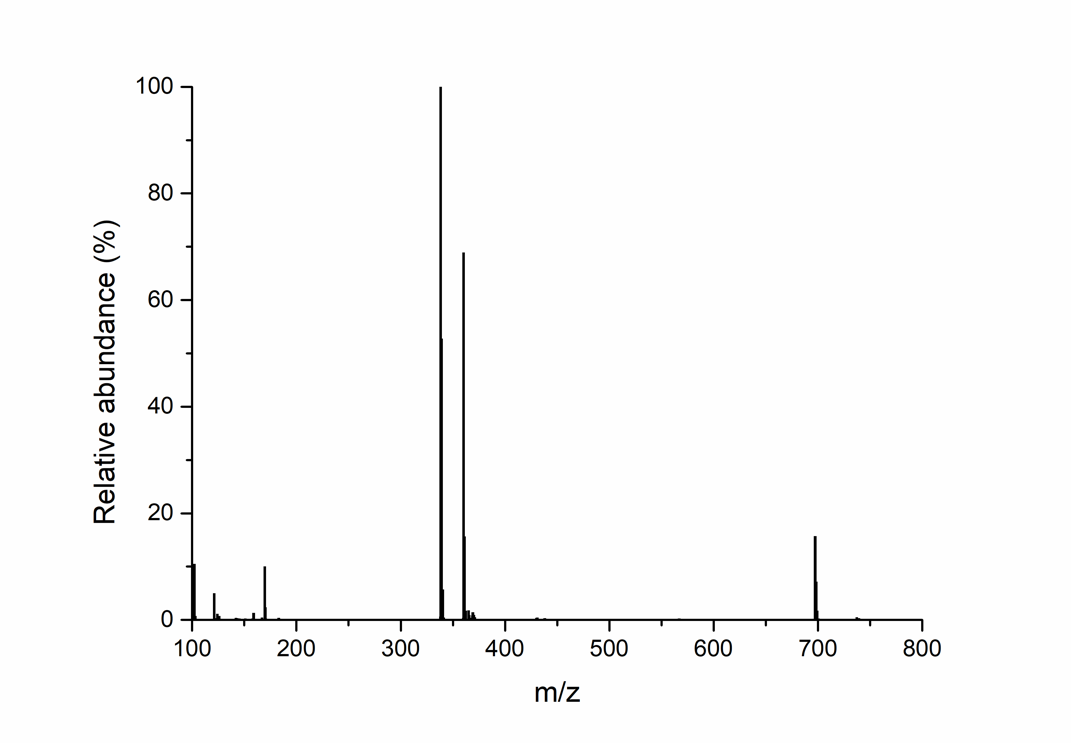


**Figure S6.** Positive mode ESI-MS of 4-[2,2';6',2'']-terpyridin-4-benzaldehyde (**2**). [M+H]^+^: 338.13.

3.3 [Ru(1)_2_](2PF_6_) (3)


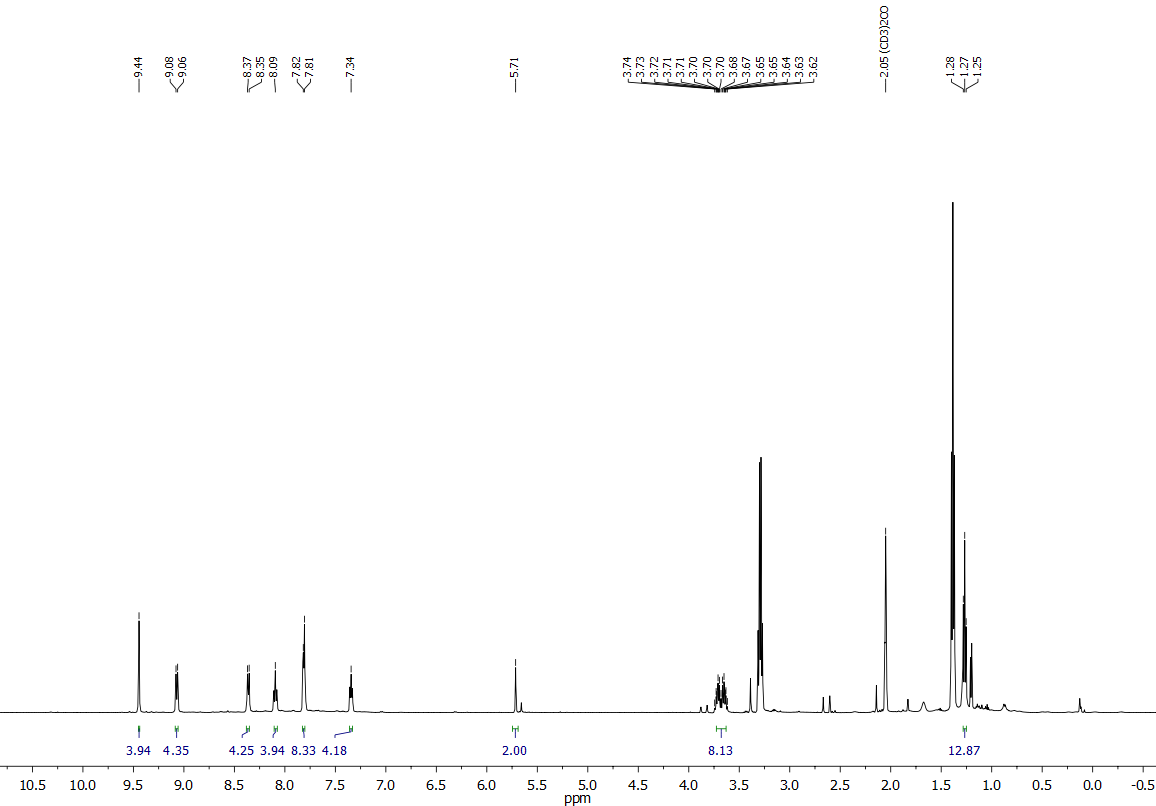


**Figure S7.** ^1^H-NMR (500 MHz, (CD_3_)_2_CO) of [Ru(**1**)_2_](2PF_6_) (**3**).


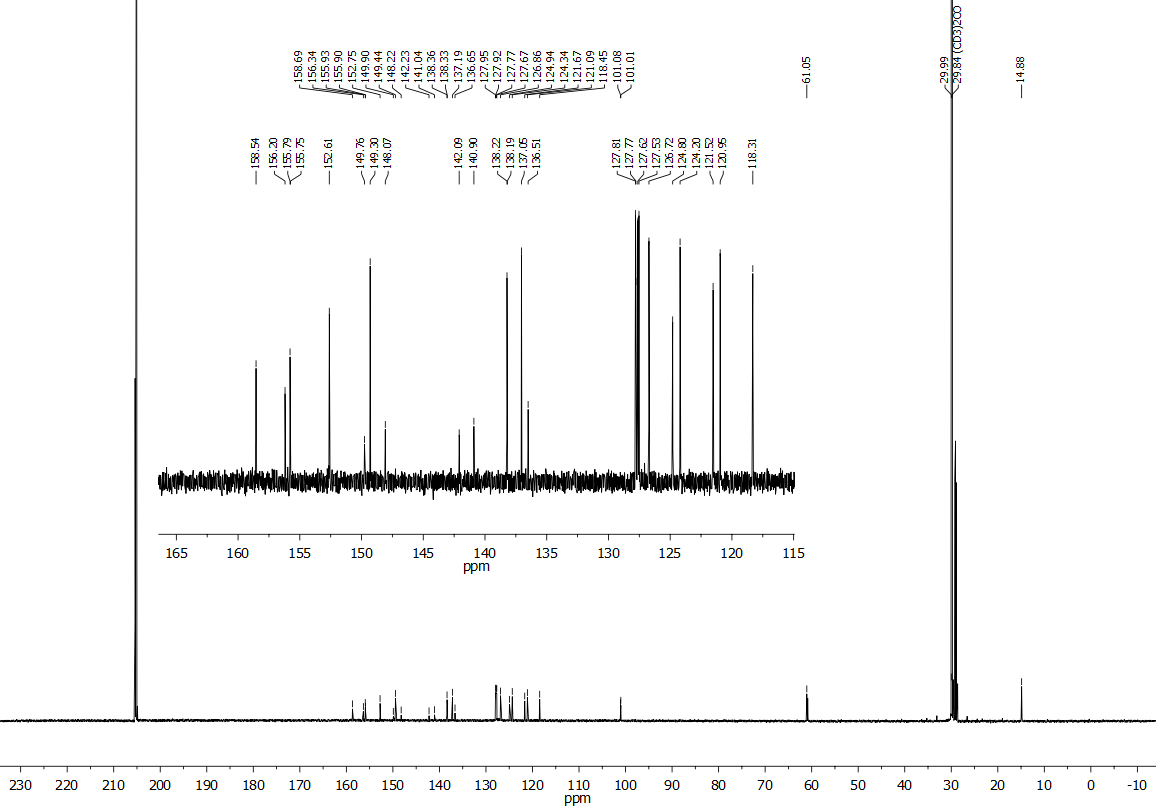


**Figure S8.** ^13^C-NMR (126 MHz, (CD_3_)_2_CO) of [Ru(**1**)_2_](2PF_6_) (**3**).


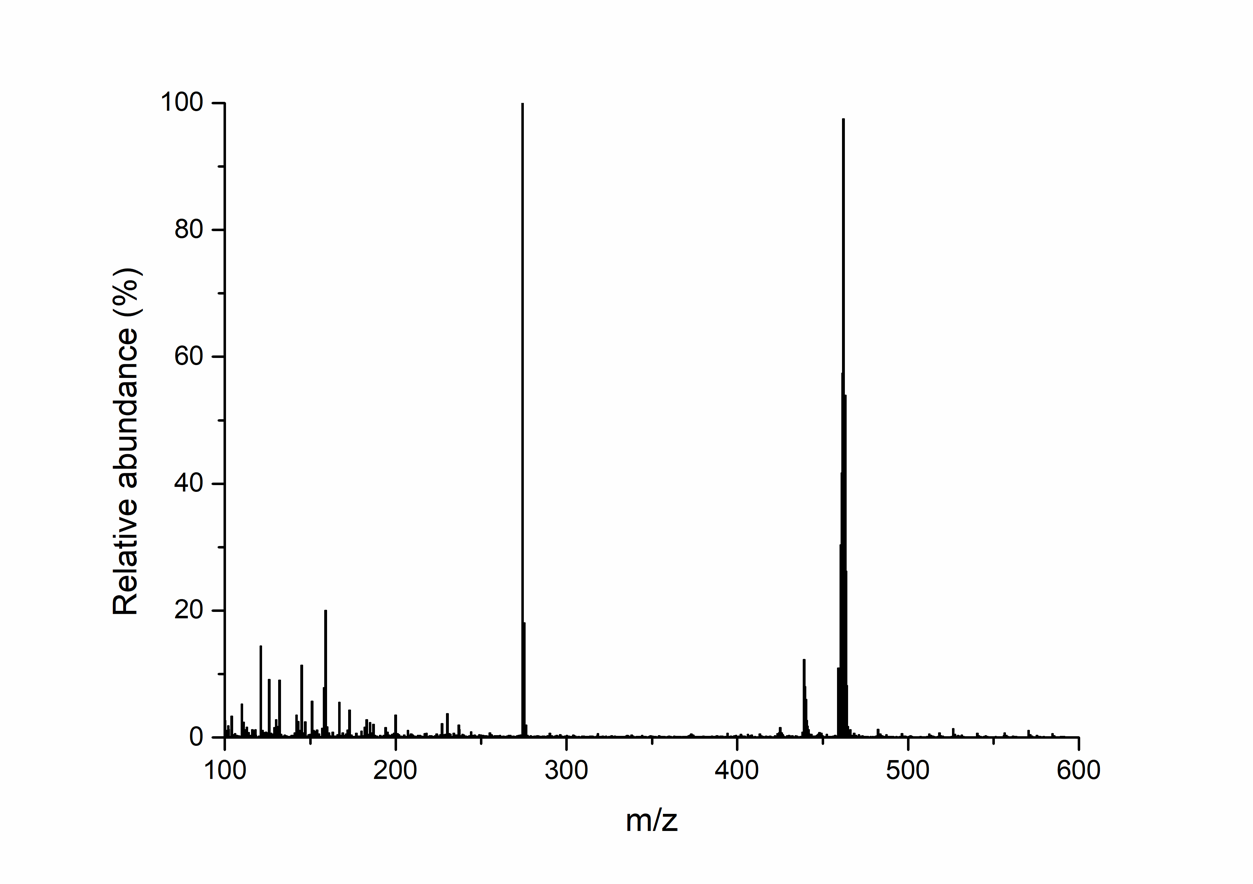


**Figure S9.** Positive mode ESI-MS of [Ru(**1**)_2_](2PF_6_) (**3**). m/z 462.89 [M/2 – 2PF_6_ + H]^+^.





**Figure S10**. UV-Vis spectra of [Ru(**1**)_2_](2PF_6_) (**3**) in CH_3_CN; ɛ_(491 nm)_ = 19800 L mol^-1^ cm^-1^.

3.4. Ru-pyrene MCOF


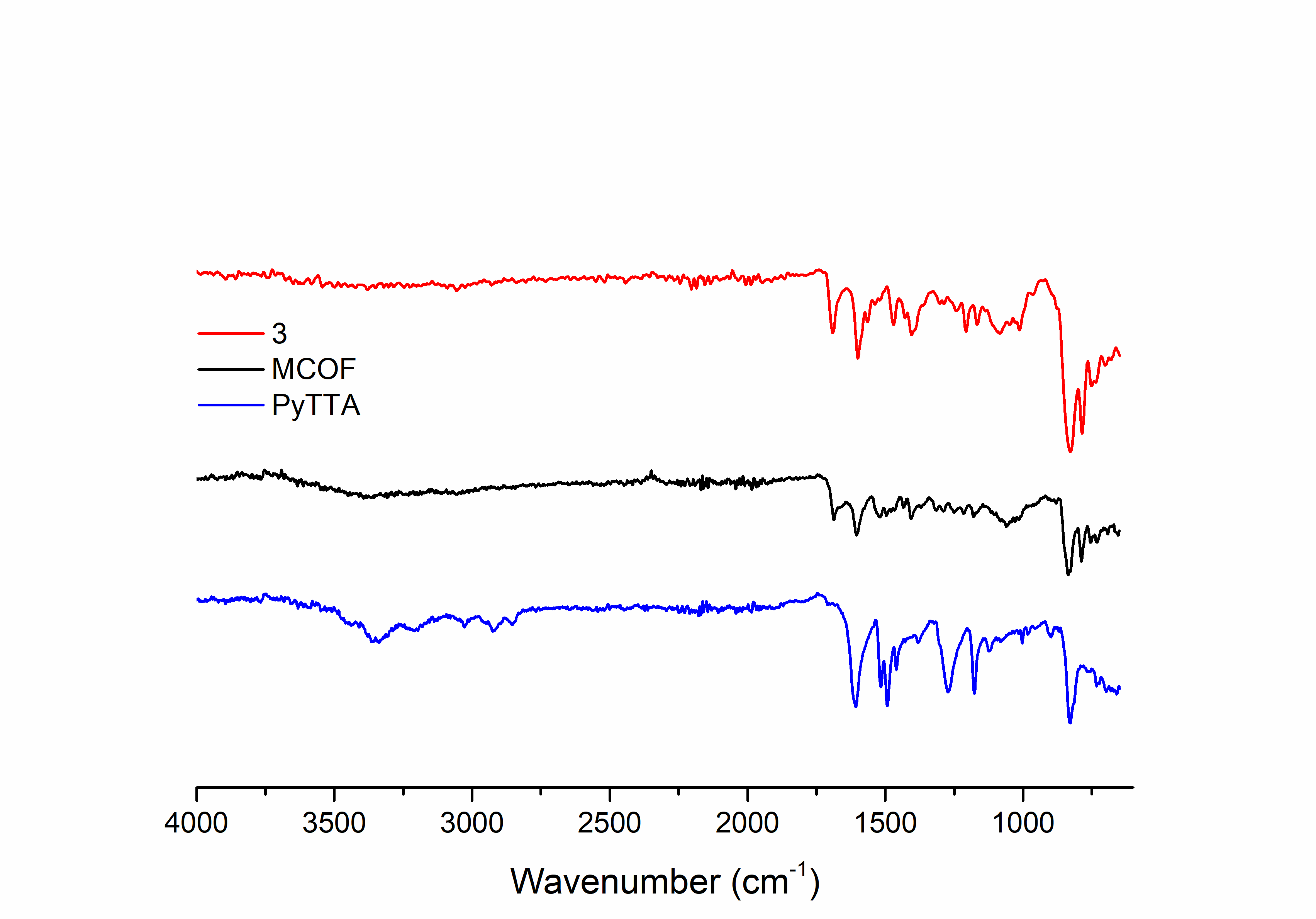


**Figure S11.** IR spectra comparisons of [Ru(**1**)_2_](2PF_6_) (**3**), Ru-pyrene MCOF and PyTTA.





**Figure S12**. Assigned regions of interest for IR spectra comparisons of [Ru(**1**)_2_](2PF_6_) (**3**), Ru-pyrene MCOF and PyTTA; a zoomed-in region (1800–650 cm^-1^) is shown for greater clarity.


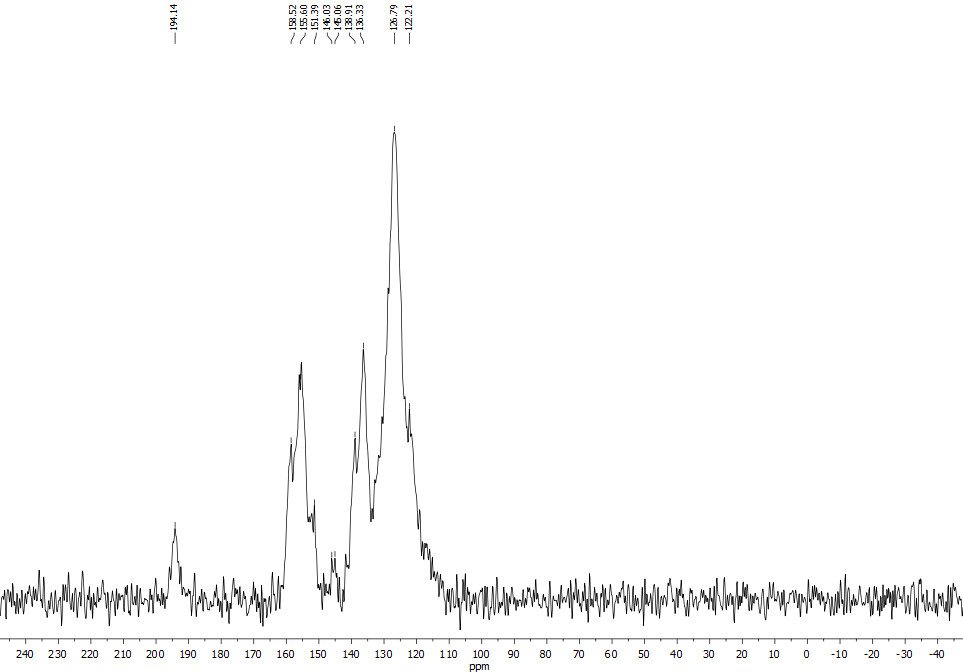


**Figure S13.** Solid-state CP-MAS ^13^C-NMR of Ru-pyrene MCOF.


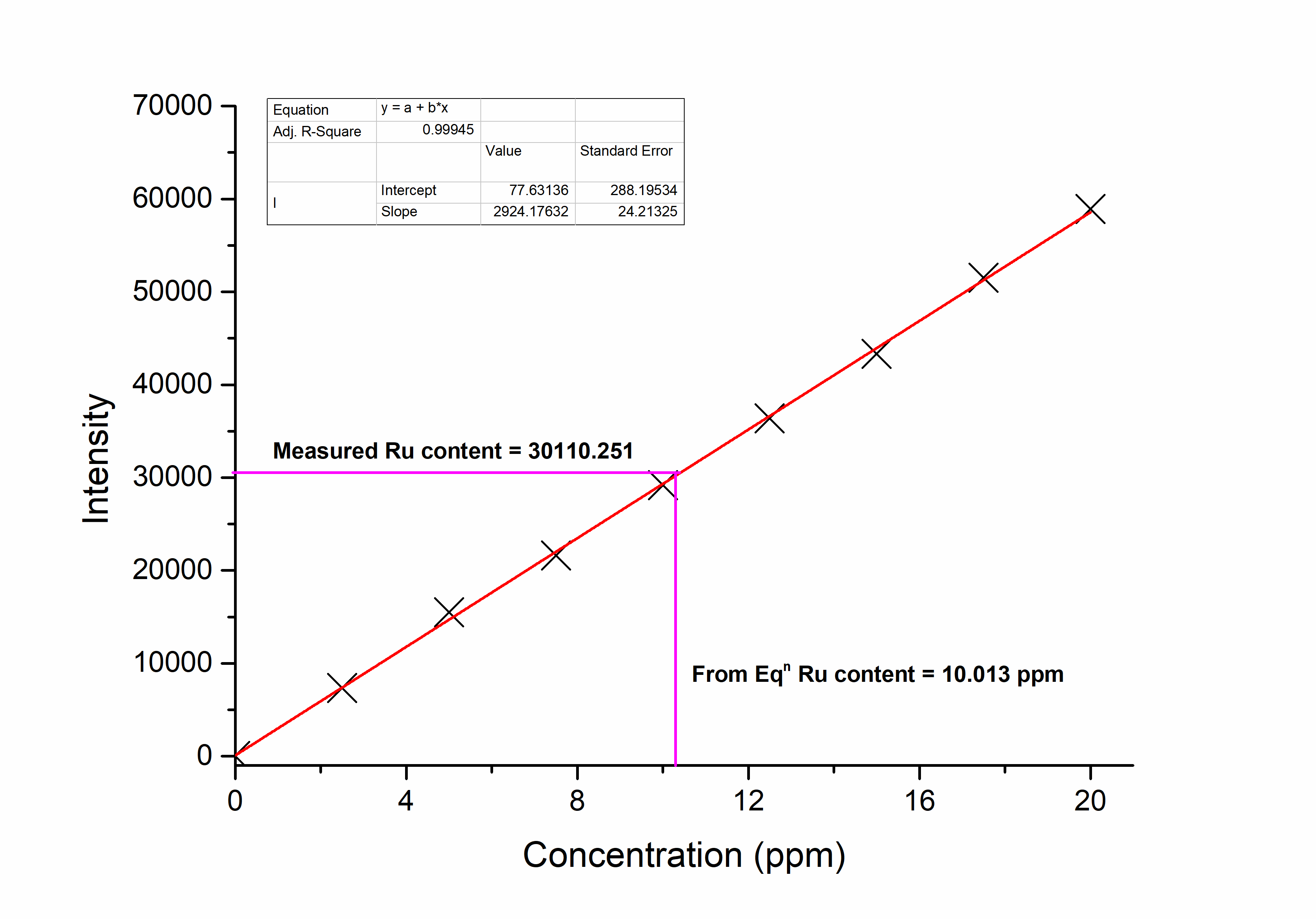


**Figure S14.** ICP-OES calibration curve; measured Ru content in Ru-pyrene MCOF is highlighted.





**Figure S15.** PXRD of [Ru(**1**)_2_](2PF_6_) (**3**), Ru-pyrene MCOF and PyTTA. The defined peaks at 37, 44, 64 and 77 (2θ) are attributed to the aluminium sample holder.^[18]^ The broad background for MCOF between 12-25 (2θ) is attributed to the polyimide tube sample holder.

**Table S16.** Diffraction angles obtained from PXRD data of Ru-pyrene MCOF, with their corresponding *d*-values.

| **2θ** | **d (Å)** |
| --- | --- |
| 3.21 | 27.50 |
| 4.16 | 21.22 |
| 5.77 | 15.30 |
| 8.34 | 10.39 |
| 10.48 | 8.43 |
| 11.85 | 7.46 |
| 13.24 | 6.68 |
| 18.06 | 4.91 |
| 20.64 | 4.30 |
| 21.81 | 4.07 |
| 22.51 | 3.94 |
|  |  |

**Figure S17.** SAXS of Ru-pyrene MCOF.


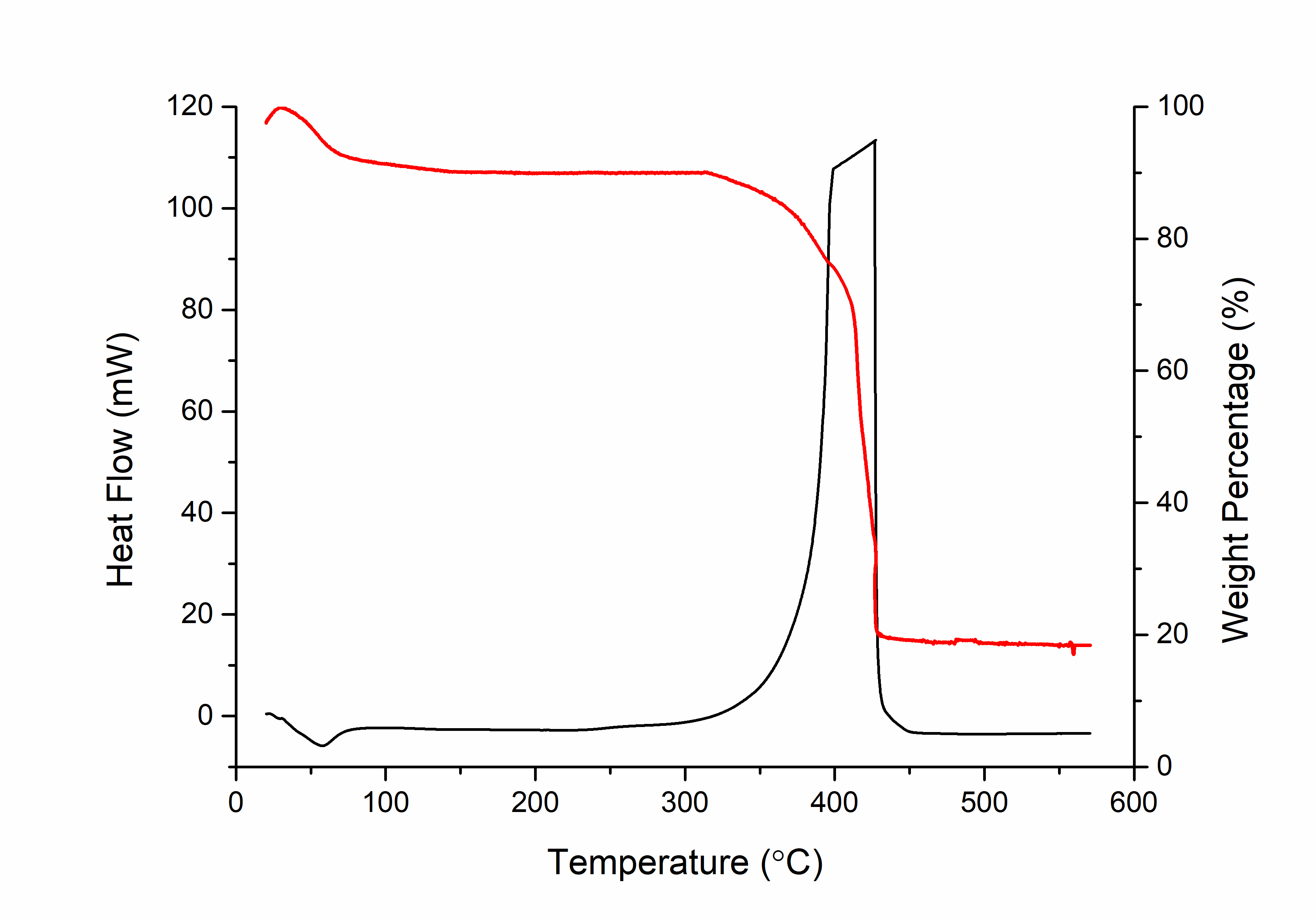


**Figure S18.** TGA-DSC of Ru-pyrene MCOF.


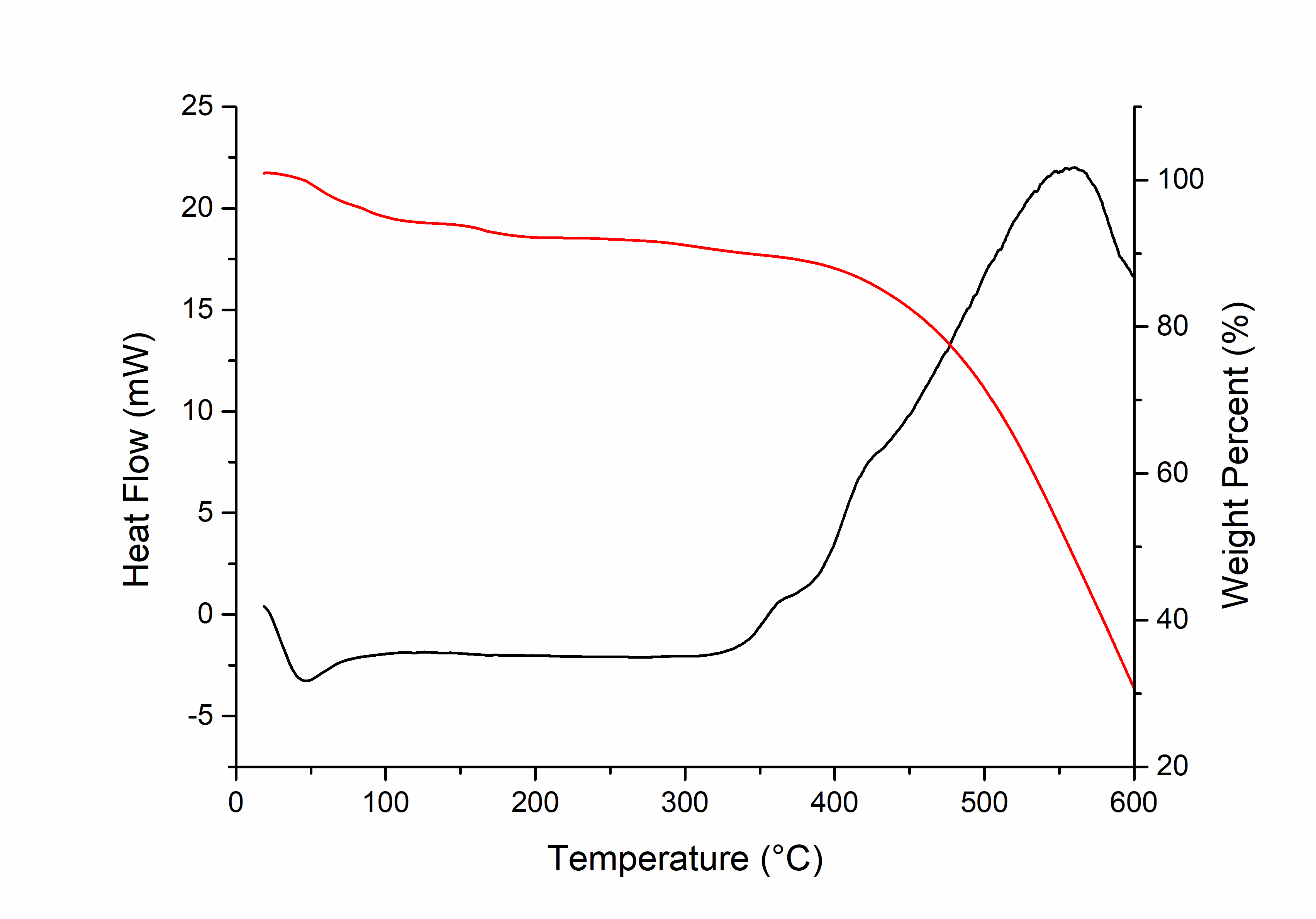


**Figure S19.** TGA-DSC of PyTTA.


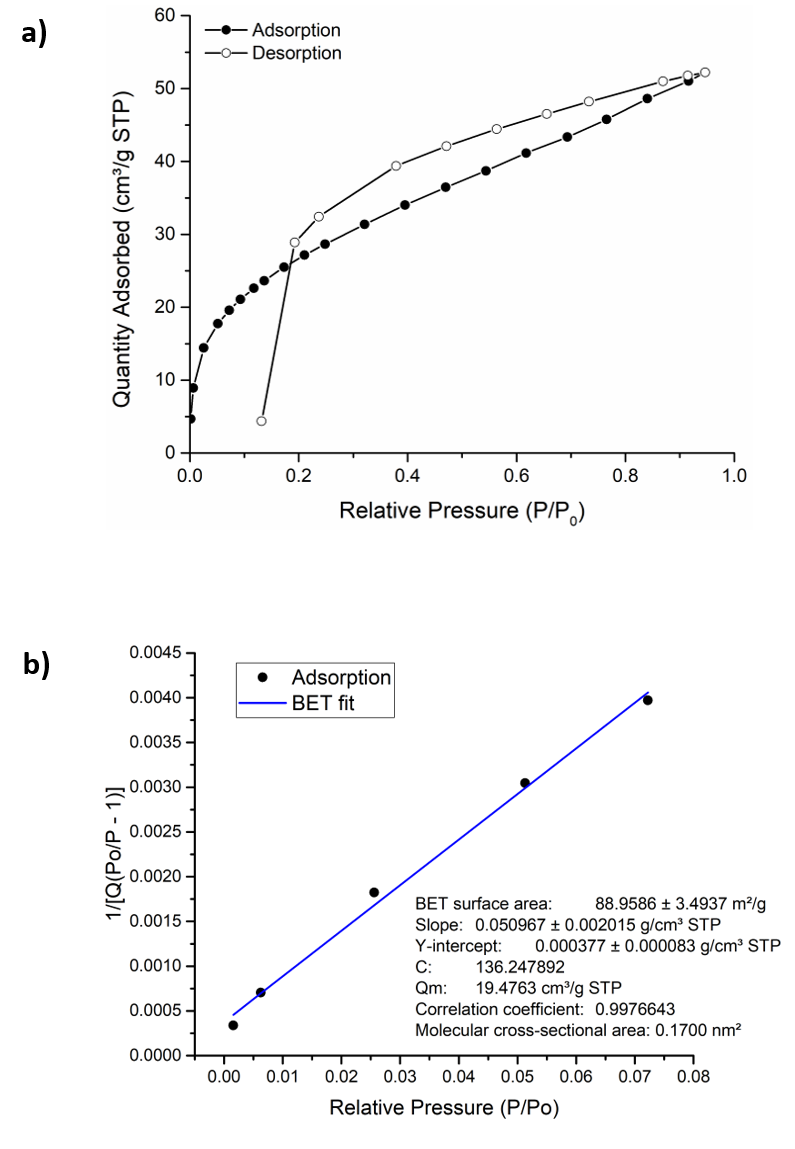


**Figure S20.** (**a**) CO_2_ 195 K sorption and (**b**) BET analysis of adsorption in the Ru-pyrene MCOF.





**Figure S21.** 100 consecutive cycle CV measurement of Ru-pyrene MCOF cast onto a glassy carbon electrode with Nafion. Measurements were performed in N_2_-bubbled dry CH_3_CN with a 0.1 M ^n^Bu₄PF₆ supporting electrolyte between 0.50 → 1.60 V_SCE­_ at a scan rate of 0.1 V s^-1^.





**Figure S22.** Emission spectra of [Ru(**1**)_2_](2PF_6_) (**3**) (red), Ru-pyrene MCOF (black) and PyTTA (blue). The bare PMMA film is also shown for comparison (green). All measurements were performed on PMMA‐dispersed films on quartz slides at λ_exc_ = 350 nm. A blue shift in the emission spectra (465 → 451 nm) of the pyrene moieties is also highlighted.


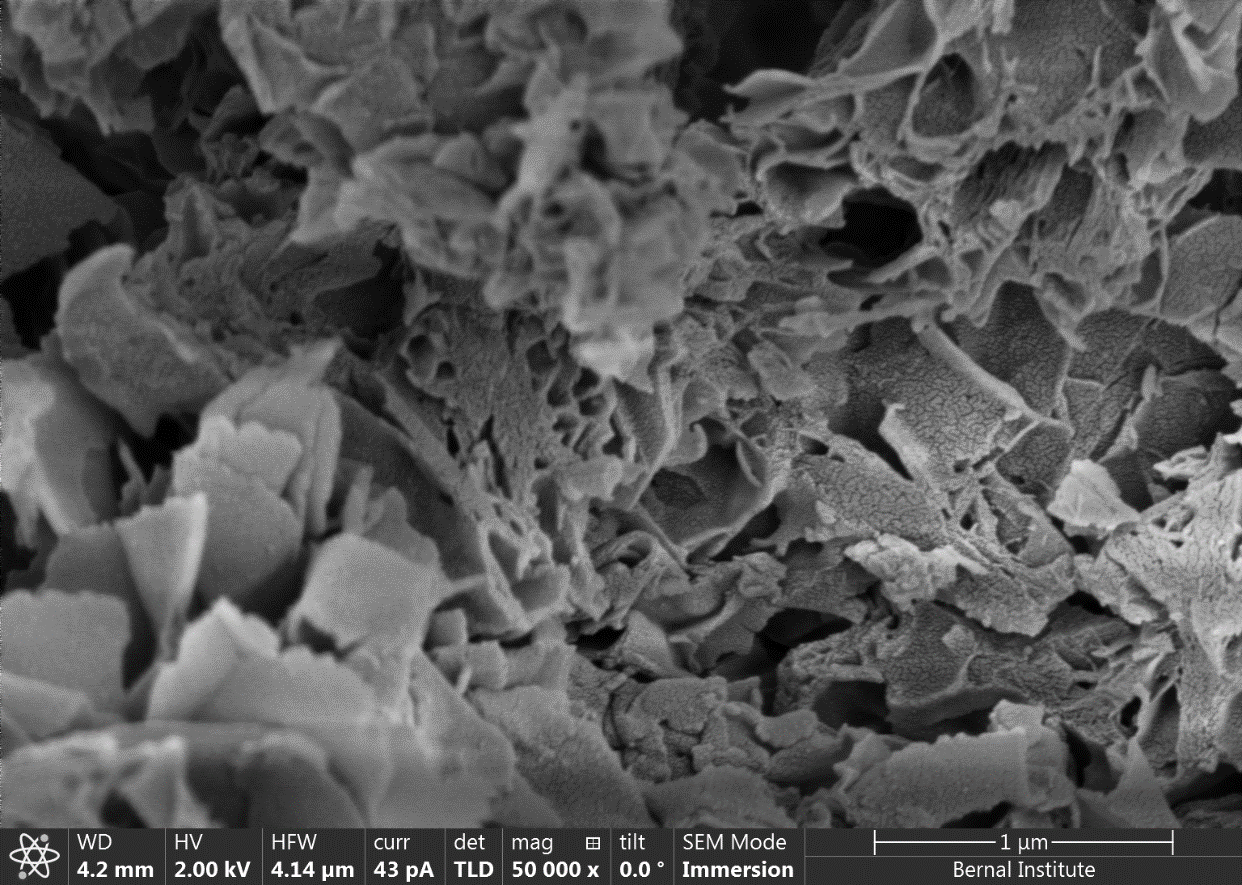


**Figure S23.** SEM image of Ru-pyrene MCOF. Consistent porous web-like appearance shown in the surface of the far-distant flakes.


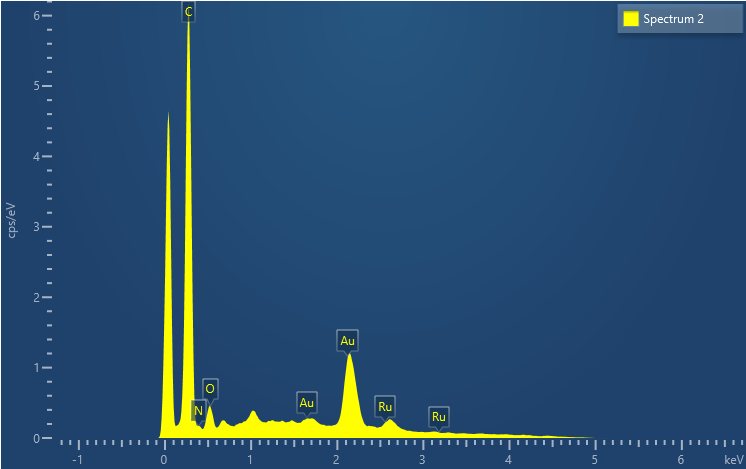


**Figure S24.** Gold-coated EDX measurement of the Ru-pyrene MCOF.


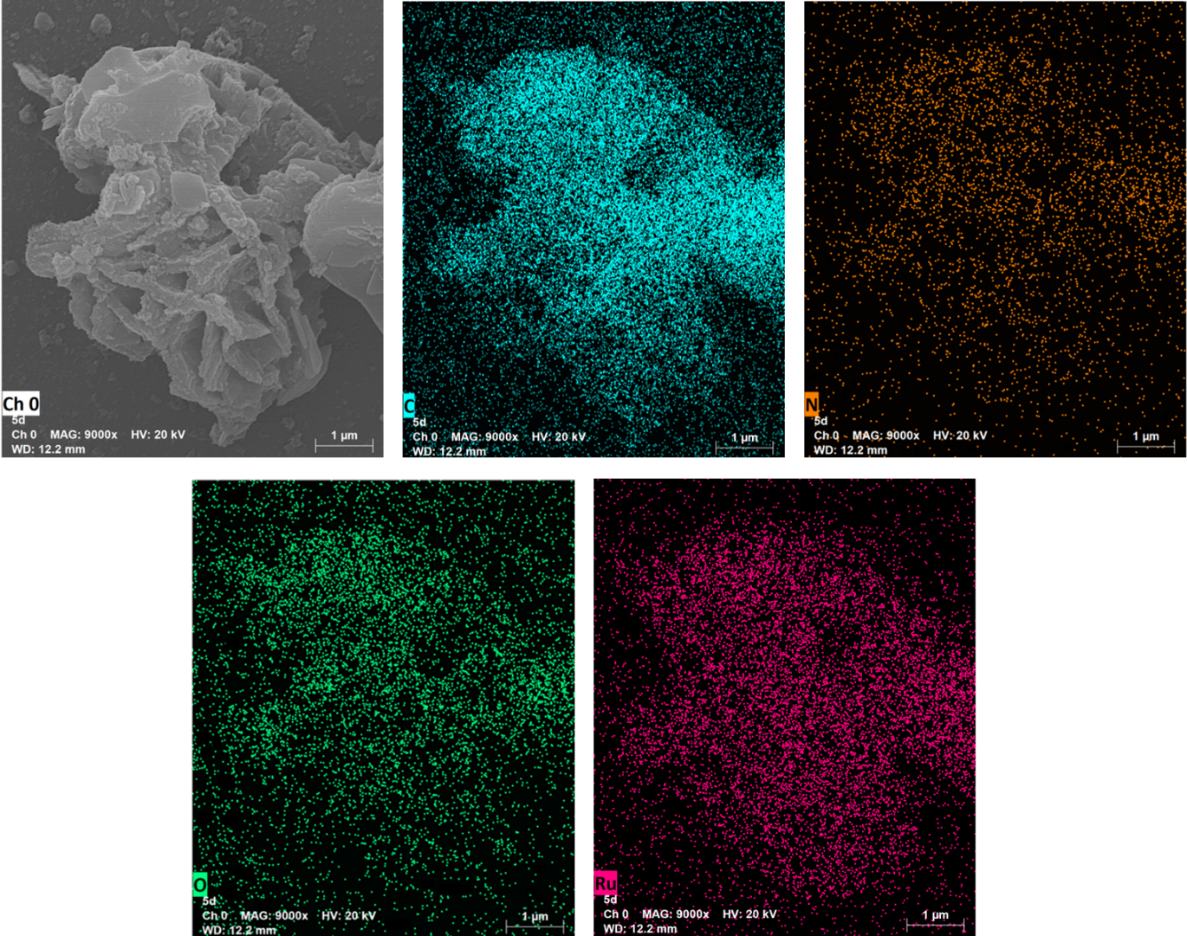


**Figure S25.** SEM-EDX mapping of carbon (blue), nitrogen (orange), oxygen (green) and ruthenium (pink) content on the Ru-pyrene MCOF.


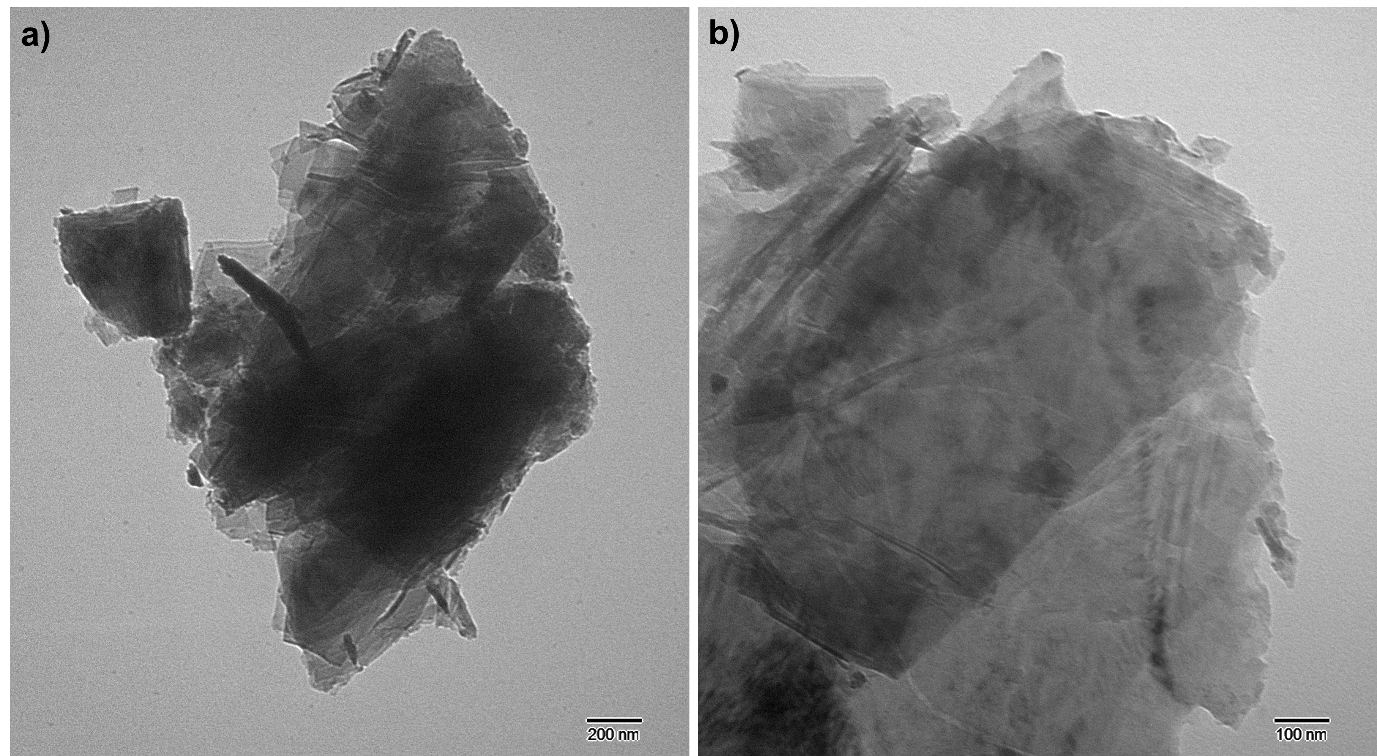


**Figure S26.** TEM imaging of a non-exfoliated Ru-pyrene MCOF sample. Multilayer aggregation can be distinguished throughout the sample.


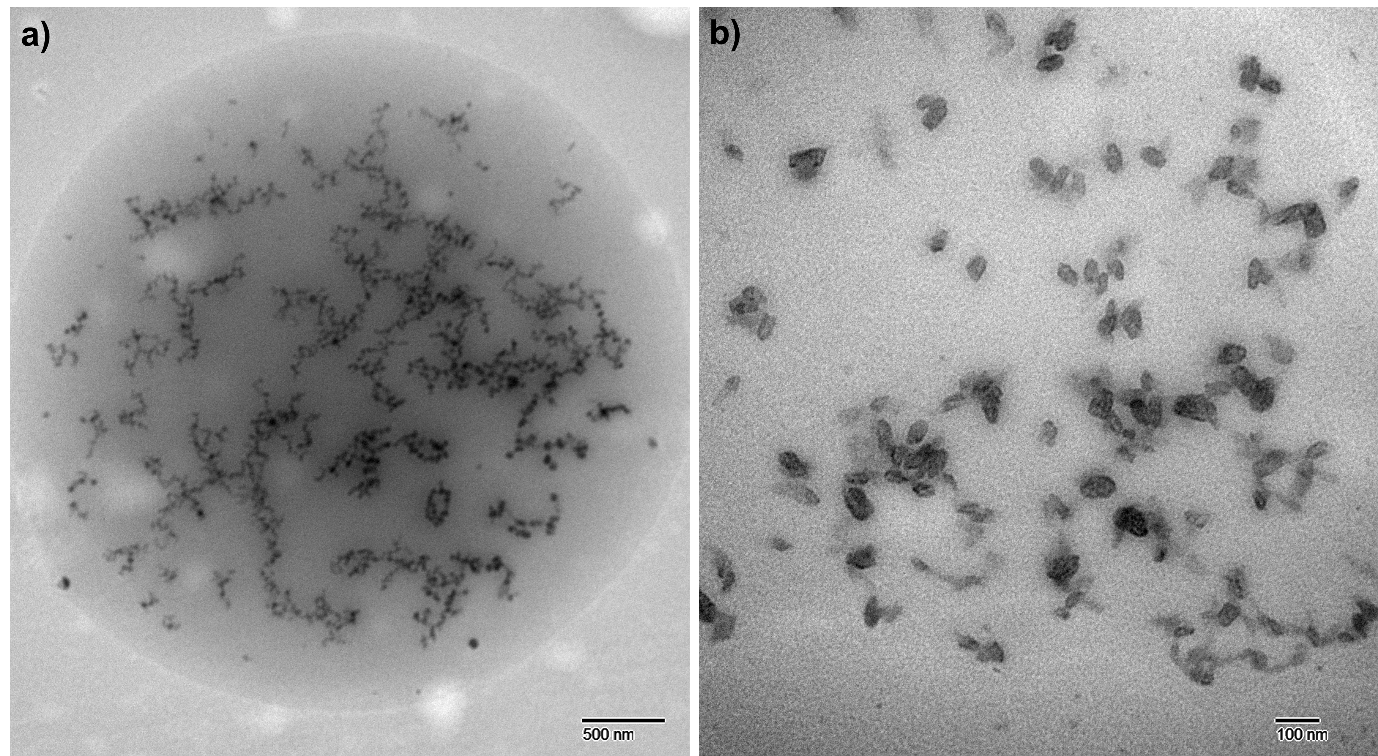


**Figure S27.** TEM imaging of an exfoliated Ru-pyrene MCOF sample. Exfoliated samples were sonicated in ethanol at 50 °C for 6 h before deposition.


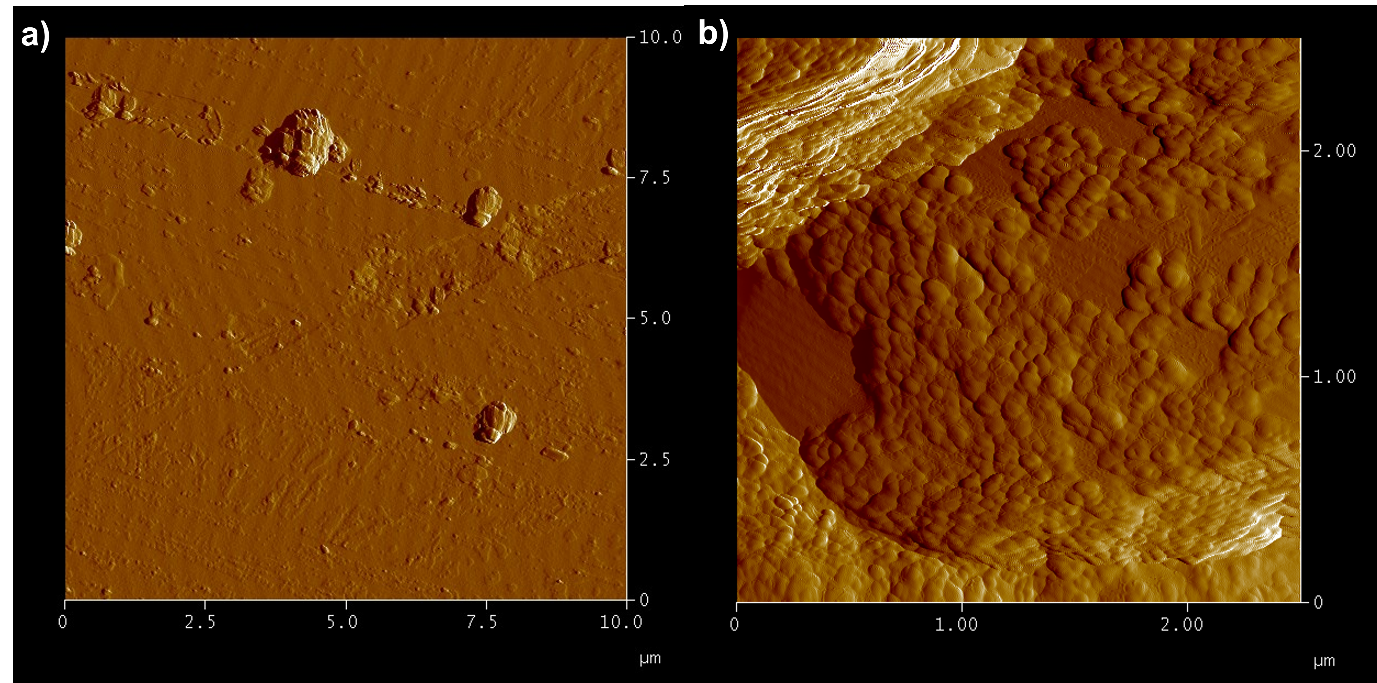


**Figure S28****.** (**a**) AFM image of the non-exfoliated sample of the Ru-pyrene MCOF. Aggregate formation of the Ru-pyrene MCOF is shown in the form of large clusters. (**b**) AFM imaging of an exfoliated MCOF sample. Exfoliated samples were sonicated in ethanol at 50 °C for 6 h before deposition.

**
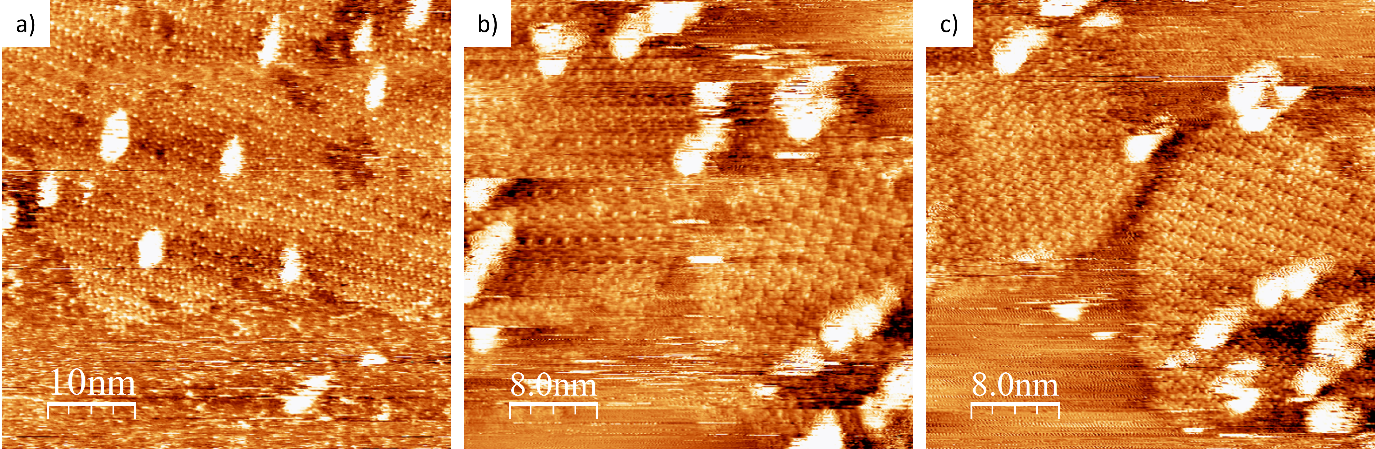
**

**Figure S29.** STM images showing a periodic pattern of bright protrusions acquired after deposition of building blocks, as well as solid particles of the Ru-pyrene MCOF at the heptanoic acid/HOPG interface. V_bias_= -0.4 V I_set_= 150 pA .


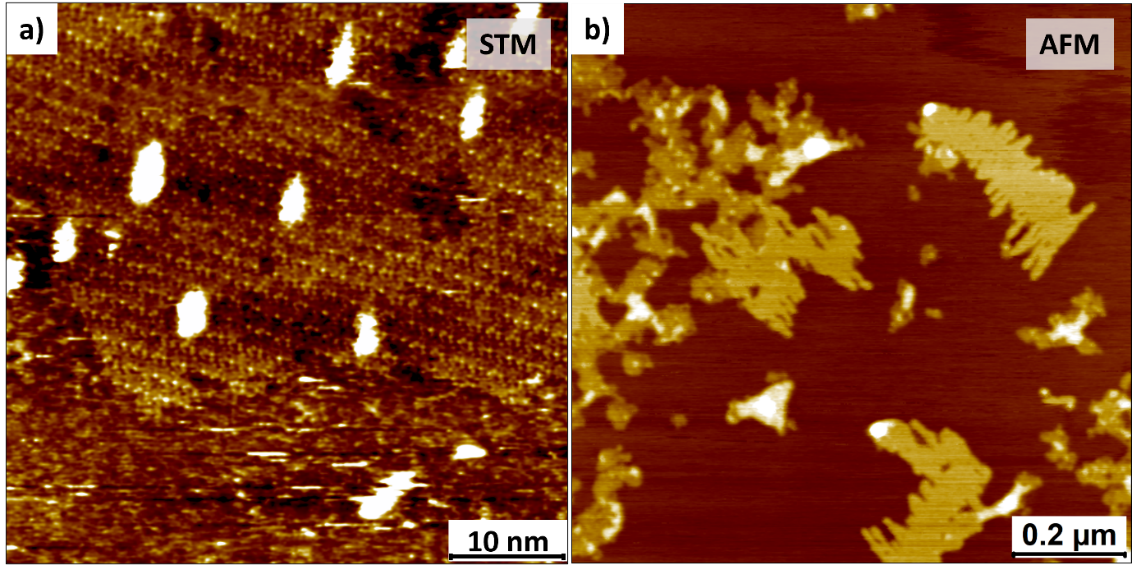


**Figure S30.** (**a**) STM image showing the boundary of the Ru-pyrene MCOF domain at the heptanoic acid/HOPG interface. V_bias_= -0.4 v I_set_= 150 pA and (**b**) AFM image showing the topography of the MCOF on the dry HOPG surface.


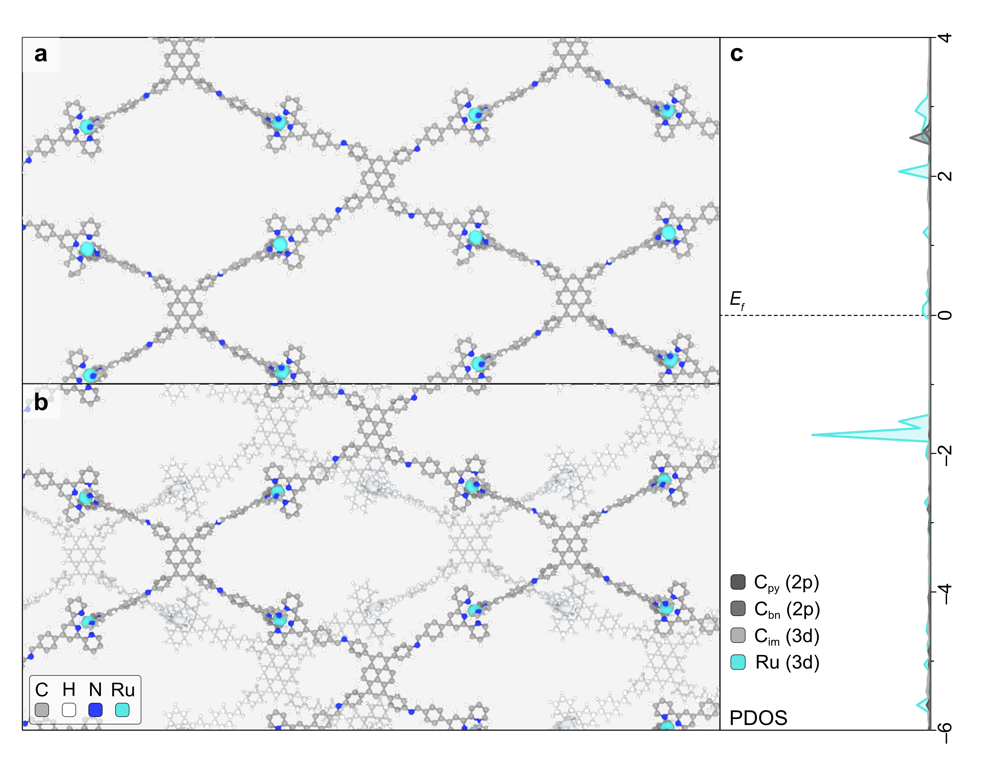


**Figure S31.** (**a**) Ru-pyrene MCOF model in the AA stacking. (**b**) Bilayer of Ru-pyrene MCOF model in the AB stacking, characterized by a shift of 15.130 Å along the *x* axis and of 3.381 Å along the *y* axis. (**c**) Calculated projected density of states (PDOS) of a single C_py_, C_bn_, C_im_ and Ru atoms belonging to the Ru-pyrene MCOF model with the AB stacking depicted in Figure 6a, with the contributions of the atomic orbitals highlighted in different colours.

**Table S32.** Vibrational frequencies, **𝜈** (in cm^–1^), computed for the Ru-pyrene complex **3** within the region of 1500 and 1650 cm^–1^.

| **𝜈** | **𝜈** |
| --- | --- |
| 1508 | 1559 |
| 1510 | 1561 |
| 1514 | 1569 |
| 1517 | 1573 |
| 1520 | 1583 |
| 1534 | 1607 |
| 1545 | 1616 |


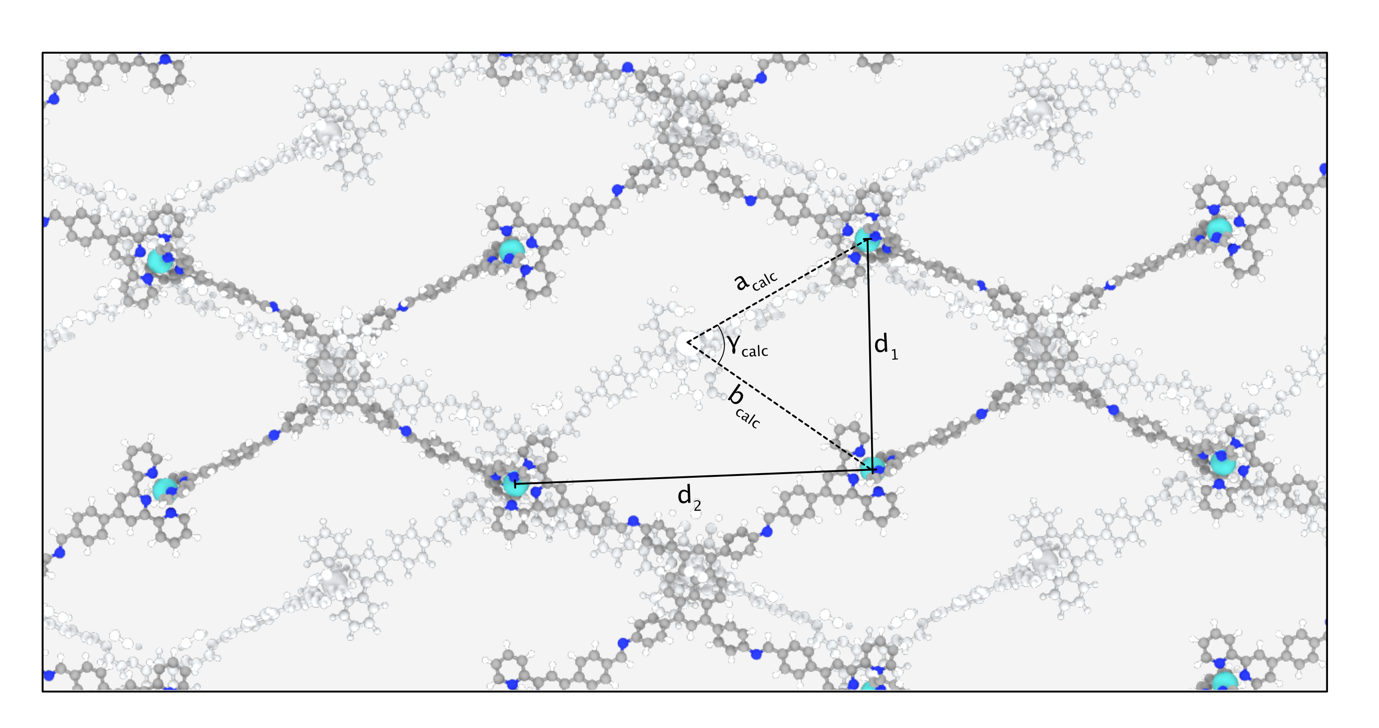


**Figure S33.** Depiction of the *a_calc_* and *b_calc_* vectors and $\gamma_{calc}$ angle. The Ru-Ru intralayer distances along $d_{1}$ and $d_{2}$, are highlighted. The values of $d_{1}$ and $d_{2}$ that would feature a $\gamma_{calc}$ angle of 80° were calculated according to the equations:

$$d_{1}=2a_{calc}\sin\left( \gamma/2 \right)$$

$$d_{2}=2a_{calc}\cos\left( \gamma/2 \right)$$

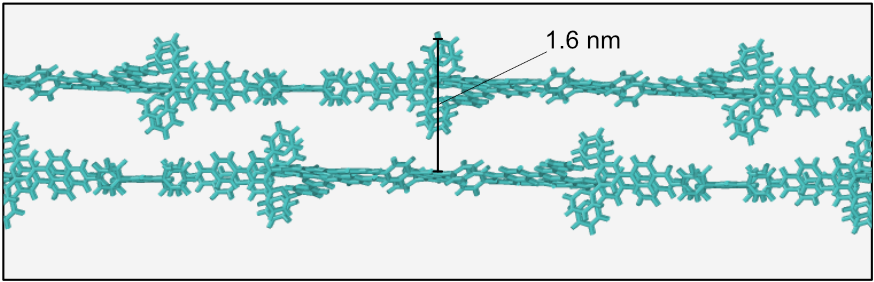


**Figure S34.** Ru-pyrene MCOF bilayer thickness for the reported model in Figure 6a. Measured as the distance between the uppermost C of a pyridine ligand from the top layer and a pyrene core of the layer underneath.

**Table S35.** Summary of the computed *c*-axis lengths and corresponding A-B interlayer distances (in Å). The spacing between the A and B layers within a unit cell is labelled as $d_{1}$, while $d_{2}$ is the spacing between the B layer within a unit cell and the A′ layer in the neighbouring periodic image along the *c*-axis. $d_{1}$ and $d_{2}$ were calculated as follows:

$$d_{1}=\left( average c_{layer B}-average c_{layer A} \right)\times c$$

$$d_{2}=\left( average c_{layer A^{'}}-average c_{layer B} \right)\times c$$

where $c$ is the length of the *c*-axis, and $average c_{layer X}$ is the average direct *c*-coordinate of the atoms in layer *X*. $\left| \Delta d \right|$ represents the absolute value of the difference (in Å) between $d_{1}$ and $d_{2}$.

| ***c*-axis** | $\boldsymbol{d}_{\boldsymbol{1}}$ | $\boldsymbol{d}_{\boldsymbol{2}}$ | $\left\vert\boldsymbol{\Delta}\boldsymbol{d} \right\vert$ |
| --- | --- | --- | --- |
| 22.274 | 10.706 | 11.568 | 0.862 |
| 22.108 | 10.706 | 11.402 | 0.695 |
| 21.941 | 10.706 | 11.235 | 0.529 |
| 21.775 | 10.706 | 11.069 | 0.363 |
| 21.609 | 10.706 | 10.902 | 0.197 |
| 21.443 | 10.706 | 10.737 | 0.031 |
| 21.277 | 10.706 | 10.570 | 0.136 |

**Table S36.** Comparison between the computed and experimental (Table S16) X-ray diffraction peaks with intensities exceeding 5%. Peaks that most closely match in terms of both 2θ values and interplanar distances are highlighted in the same colour. In each grouping, the most intense peak is accentuated in bold.

| **Experimental spectrum** | **Computed**  **spectrum** | |  |
| --- | --- | --- | --- |
| **2θ** | **2θ** | **I (%)** | $\left\vert\boldsymbol{\Delta}\boldsymbol{2}\boldsymbol{\theta} \right\vert$ |
| 3.21° | **2.49°** | **100.00** | **0.72** |
| 4.16° | **4.61°** | **28.41** | **0.45** |
|  | 4.92° | 18.52 | 0.76 |
| 5.77° | **4.97°** | **93.83** | **0.80** |
|  | 5.00° | 16.77 | 0.77 |
|  | 5.06° | 88.02 | 0.71 |
|  | 5.10° | 20.67 | 0.67 |
|  | 5.41° | 26.42 | 0.36 |
|  | 5.54° | 32.08 | 0.23 |
|  | 6.28° | 24.82 | 0.51 |
| 8.34° | **8.31°** | **53.44** | **0.03** |
|  | 8.44° | 8.25 | 0.10 |
|  | 9.22° | 9.79 | 0.88 |
|  | 9.27° | 11.26 | 0.93 |
|  | 9.32° | 6.12 | 0.98 |
|  | 9.37° | 5.00 | 1.03 |
| 10.48° | 9.95° | 5.21 | 0.53 |
|  | 10.09° | 5.96 | 0.39 |
|  | **10.12°** | **5.99** | **0.36** |
|  | 10.18° | 5.03 | 0.30 |


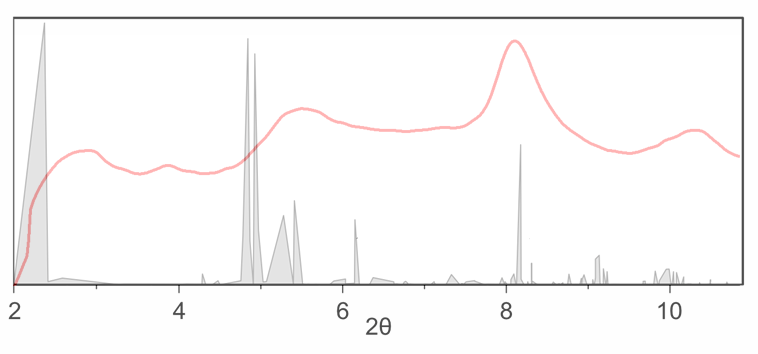


**Figure S37.** Simulated XRD (grey) from DFT calculations, peaks in computed diffractogram is dictated by the "simplified" model of the unit cell, overlapped with experimental data (red, baseline uncorrected), from 2–11 (2θ).

**Table S38.** Comparison of the first diffraction peak obtained by experimental PXRD (Table S16) and computational modelling. Stacked layers were shifted in the models to produce disorder in the structure and evaluate changes in peak positions and relative intensities.

|  | **Disorder**  **applied** | **2θ** | **I (%)** |
| --- | --- | --- | --- |
| **Experimental spectrum** | --- | 3.21° |  |
| **Computed Spectra** | Non-applied | 2.49° | 100.00 |
|  | AA | 2.71° | 100.00 |
|  | AB 1D  “x” shift | 2.49° | 47.00 |
|  | AB 1D  “z” shift | 2.70° | 100.00 |
|  | AB diagonal  asymmetric shift | 2.52° | 80.00 |
|  | AB diagonal  symmetric shift | 2.49° | 100.00 |
|  | ABC | 2.52° | 65.00 |
|  | ABCD | 2.52° | 90.00 |
|  | ABCDE | 2.52 | 64.00 |

# References

[1] R. Kilaas, Optimal and near-optimal filters in high-resolution electron microscopy. *J. Microsc.*, **1998**, *190*, 45. DOI: 10.1046/j.1365-2818.1998.3070861.x.

[2] G. Kresse, J. Furthmüller, Efficient iterative schemes for ab initio total-energy calculations using a plane-wave basis set. *Phys. Rev. B*, **1996**, *54*, 11169. DOI: 10.1103/PhysRevB.54.11169.

[3] J. P. Perdew, K. Burke, M. Ernzerhof, Generalized Gradient Approximation Made Simple. *Phys. Rev. Lett.*, **1996**, *77*, 3865. DOI: 10.1103/PhysRevLett.77.3865.

[4] S. Grimme, J. Antony, S. Ehrlich, H. Krieg, A consistent and accurate ab initio parametrization of density functional dispersion correction (DFT-D) for the 94 elements H-Pu. *J. Chem. Phys.*, **2010**, *132*, 154104. DOI: 10.1063/1.3382344.

[5] P. E. Blöchl, Projector augmented-wave method. *Phys. Rev. B*, **1994**, *50*, 17953. DOI: 10.1103/PhysRevB.50.17953.

[6] D. D. Johnson, Modified Broyden’s method for accelerating convergence in self-consistent calculations. *Phys. Rev. B*, **1988**, *38*, 12807. DOI: 10.1103/PhysRevB.38.12807.

[7] A. Hjorth Larsen, J. Jørgen Mortensen, J. Blomqvist, I. E. Castelli, R. Christensen, M. Dułak, J. Friis, M. N. Groves, B. Hammer, C. Hargus, E. D. Hermes, P. C. Jennings, P. Bjerre Jensen, J. Kermode, J. R. Kitchin, E. Leonhard Kolsbjerg, J. Kubal, K. Kaasbjerg, S. Lysgaard, J. Bergmann Maronsson, T. Maxson, T. Olsen, L. Pastewka, A. Peterson, C. Rostgaard, J. Schiøtz, O. Schütt, M. Strange, K. S. Thygesen, T. Vegge, L. Vilhelmsen, M. Walter, Z. Zeng, K. W. Jacobsen, The atomic simulation environment—a Python library for working with atoms. *J. Phys. Condens. Matter.*, **2017**, *29*, 273002. DOI: 10.1088/1361-648X/aa680e.

[8] M. P. Teter, M. C. Payne, D. C. Allan, Solution of Schrödinger’s equation for large systems. *Phys. Rev. B*, **1989**, *40*, 12255. DOI: 10.1103/PhysRevB.40.12255.

[9] P. E. Blöchl, O. Jepsen, O. K. Andersen, Improved tetrahedron method for Brillouin-zone integrations. *Phys. Rev. B*, **1994**, *49*, 16223. DOI: 10.1103/PhysRevB.49.16223.

[10] A. M Ganose, A. J Jackson, D. O Scanlon, sumo: Command-line tools for plotting and analysis of periodic ab initio calculations. *J. Open Source Softw.*, **2018**, *3*, 717. DOI: 10.21105/joss.00717.

[11] Y. Le Page, P. Saxe, Symmetry-general least-squares extraction of elastic data for strained materials from ab initio calculations of stress. *Phys. Rev. B*, **2002**, *65*, 104104. DOI: 10.1103/PhysRevB.65.104104.

[12] P. Pulay, Convergence acceleration of iterative sequences. the case of scf iteration. *Chem. Phys. Lett.*, **1980**, *73*, 393. DOI: 10.1016/0009-2614(80)80396-4.

[13] S. Leung, https://github.com/ShuangLeung/STM_2DScan.git. **2020**.

[14] K. Momma, F. Izumi, VESTA 3 for three-dimensional visualization of crystal, volumetric and morphology data. *J. Appl. Crystallogr.*, **2011**, *44*, 1272. DOI: 10.1107/S0021889811038970.

[15] E. Dulière, M. Devillers, J. Marchand-Brynaert, Novel phosphinite-ruthenium(II) complexes covalently bound on silica: Synthesis, characterization, and catalytic behavior versus oxidation reactions of alcohols into aldehydes. *Organometallics*, **2003**, *22*, 804. DOI: 10.1021/om020876+.

[16] F. Auras, L. Ascherl, A. H. Hakimioun, J. T. Margraf, F. C. Hanusch, S. Reuter, D. Bessinger, M. Döblinger, C. Hettstedt, K. Karaghiosoff, S. Herbert, P. Knochel, T. Clark, T. Bein, Synchronized Offset Stacking: A Concept for Growing Large-Domain and Highly Crystalline 2D Covalent Organic Frameworks. *J. Am. Chem. Soc.*, **2016**, *138*, 16703. DOI: 10.1021/jacs.6b09787.

[17] E. C. Constable, C. E. Housecroft, S. L. Kokatam, E. A. Medlycott, J. A. Zampese, Fe-only hydrogenase active site mimics: Fe_2_(CO)_6_(μ-ADT) (ADT=azadithiolate) clusters bearing pendant 2,2′:6′,2″-terpyridine domains and containing alkynylthienylene or alkynylphenylene spacers. *Inorg. Chem. Commun.*, **2010**, *13*, 457. DOI: 10.1016/j.inoche.2009.06.003.

[18] H. Zhang, N. Maljkovic, B. S. Mitchell, Structure and interfacial properties of nanocrystalline aluminum/mullite composites. *Mater. Sci. Eng. A*, **2002**, *326*, 317. DOI: 10.1016/S0921-5093(01)01500-3.

# Author Contributions

Seán Hennessey – writing of original draft, synthesis of all materials.

Roberto González-Gómez – data curation, synthesis of materials, analytical investigations, lead project administration.

Nicolás Arisnabarreta – data curation, analytical investigations.

Anna Ciotti – computational investigations

Jing Hou and Nadezda V. Tarakina – data curation, analytical investigations.

Kunal S. Mali – analytical investigations.

Andrey Bezrukov and Michael Zaworotko – data curation, analytical investigations.

Steven De Feyter – funding acquisition, project administration.

Max García-Melchor – computational investigations, lead project administration

Pau Farràs – funding acquisition, lead project administration.
